# Genetic screens reveal novel major and minor players in magnesium homeostasis of *Staphylococcus aureus*

**DOI:** 10.1371/journal.pgen.1008336

**Published:** 2019-08-15

**Authors:** Emilie Trachsel, Peter Redder, Patrick Linder, Joshua Armitano

**Affiliations:** 1 Department of Microbiology and Molecular Medicine, CMU, Faculty of Medicine, University of Geneva, Geneva, Switzerland; 2 LMGM UMR5100, Centre de Biologie Integrative, Paul Sabatier University, Toulouse, France; Universidad de Sevilla, SPAIN

## Abstract

Magnesium is one of the most abundant metal ions in living cells. Very specific and devoted transporters have evolved for transporting Mg^2+^ ions across the membrane and maintain magnesium homeostasis. Using genetic screens, we were able to identify the main players in magnesium homeostasis in the opportunistic pathogen *Staphylococcus aureus*. Here, we show that import of magnesium relies on the redundant activity of either CorA2 or MgtE since in absence of these two importers, bacteria require increased amounts of magnesium in the medium. A third CorA-like importer seems to play a minor role, at least under laboratory conditions. For export of magnesium, we identified two proteins, MpfA and MpfB. MpfA, is the main actor since it is essential for growth in high magnesium concentrations. We show that gain of function mutations or overexpression of the minor factor, MpfB, which is part of a sigmaB controlled stress response regulon, can compensate for the absence of MpfA.

## Author summary

Magnesium is the most abundant metal ion in cells, yet to grow they still need to maintain its concentration within acceptable parameters relying on transporters capable of importing or exporting magnesium. This is essential to allow cells to strive in varying conditions, whether the environment is rich in magnesium, such as bones or kidneys, or poor in magnesium. Here, using various genetics screens, we identified the main transporters involved in this process in the opportunistic pathogen *Staphylococcus aureus*. Surprisingly, magnesium homeostasis had never been properly studied in this bacterium, while it is key in allowing *S*. *aureus* to colonize many different organs. We show that import and export are two independent systems. Two transporters, of two different well characterized families, MgtE and CorA, are necessary for import. Export of magnesium is mediated by MpfA, a distant homolog of a mammalian CNNM family of magnesium transport mediators.

## Introduction

Magnesium is ubiquitous in living cells, as it is a cofactor for hundreds of enzymes, essential for ribosome function, and interacts very strongly with nucleic acids (RNA, DNA and (d)NTPs) [1]. Moreover, in physiological conditions, ATP is always bound to magnesium [2]. The Mg^2+^ ion is present in large quantity, around 100 mM total in a bacterial cell, of which only an estimated 1 mM is present as free Mg^2+^ [3]. Magnesium is a particular ion because its radius changes drastically whether it is hydrated or not: the hydrated radius is ~400 times larger than its dehydrated one [4]. Due to this hydration, it cannot diffuse freely through the membranes meaning that magnesium transporters must be able to recognize the hydrated magnesium, remove the hydration shell and let the dehydrated magnesium enter the cell. In other words, they have to be specific for magnesium [3,5]. Since Mg^2+^ is so important to life and needed in such high quantity, bacteria have developed mechanisms capable of importing magnesium very efficiently even against the concentration gradient. Four types of import transporters have been described so far, CorA, MgtA/B, MgtE and a Nramp-related transporter [6,7]. Additionally, genetic screens have revealed CorB and CorC in *Salmonella* Typhimurium, which have been described as accessory proteins to CorA [8].

CorA transporters are ubiquitous in Bacteria and Archaea and display a highly conserved motif (YGMNF), essential for the selective transport of Mg^2+^ [9–11]. They belong to a larger family of metal transporter called the 2-TM-GxN proteins that are present in all domains of life [12]. CorA proteins are capable of transporting Mg^2+^, Co^2+^ and Ni^2+^ but the latter is transported with such low affinity that it seems unlikely to be physiologically relevant [13]. CorA, whose monomer is composed of a large N-terminal part located in the cytosol and two transmembrane (TM) domains located in the C-terminal part, assembles in the membrane as a cone-shaped homopentamer [14,15]. The YGMNF motif is located just after the first TM domain on the external-side, facing the central part of the pore, explaining its importance in the selectivity of the transporter [16]. Two crystal structures have been reported for the CorA family: *Thermotoga maritima* (TmCorA) and *Methanocaldococcus jannaschii* (MjCorA). Although these two proteins show a low sequence similarity (24% identity), they display a highly conserved structural layout [5]. Overall, the CorA family is characterised by low sequence similarity but high conservation of structure and function, since an archaeal or even a eukaryotic CorA-like protein can complement loss of CorA in *E*. *coli* or *S*. Typhimurium [17–19].

MgtE is part of a class of Mg^2+^ transporters first described in the Gram-positive *Bacillus firmus* [20]. It has since been crystallised and described in many other organisms [5,21]. The vertebrate homologues of MgtE (SLC41 family) have also been described as Mg^2+^ transporters [22]. MgtE senses internal Mg^2+^ concentration through its pair of CBS domains (Cystathionine Beta Synthase), domains that can also sense ATP levels, and the activity of MgtE appears unaffected by the external Mg^2+^ concentration [23–25]. MgtE has been described as the main Mg^2+^ transporter of *Bacillus subtilis*, with an additional marginal role for YfjQ (a CorA-like protein) [26].

In mammals, the CNNM (cyclin M) family encompasses four transporters sharing similarity to the poorly studied bacterial CorC family [27]. Members of this family have been shown to be involved in magnesium homeostasis and associated with important physiological mechanisms including magnesium excretion in the kidney and intestine and oncogeny through their interaction with PRLs (Phosphatases of regenerating liver) [28–30]. Whether CNNMs are actual transporters of Mg^2+^ or regulators of homeostasis is still a subject of debate [31,32].

*Staphylococcus aureus* is an opportunistic pathogen capable of growing in very diverse conditions. It can survive on surfaces, thus being a very potent source of nosocomial infections, but also on the skin or in the nasopharynx of about 30% of the population and can lead to a wide variety of infections from benign to deadly [33–35]. Among these diseases, bone infections remain some the hardest to cure due in part to the poor antibiotic bioavailability, the apparition of resistant strains during the antibiotic treatment and the high bacteria counts in these organs [36]. Indeed bones are magnesium-rich reservoirs favouring chronic *S*. *aureus* infections [37]. The Lopez Lab recently showed that growth in high magnesium concentrations can cause an *S*. *aureus* population to split into specialized cell types explaining the differences between acute and chronic infections [38]. Additionally they previously showed that high magnesium concentrations, such as that found in bones, are responsible for the appearance of strains with intermediate resistance to the antibiotic vancomycin (VISA: Vancomycin Intermediate *Staphylococcus aureus)* [36], thus highlighting the importance of magnesium in the life of *S*. *aureus* and its clinical consequences.

The *S*. *aureus* genome encodes three putative magnesium importers: *mgtE* (SA0867), *corA* (SA2137) and a *corA* paralog (SA2166, designated here as *corA2*). Moreover, we previously described a novel element involved in magnesium homeostasis, *mpfA* (Magnesium Protection Factor A—SA0657) and its paralog SA0780. We identified MpfA in a genetic screen for mutations suppressing the slow growth on defined medium of a DEAD-box helicase mutant (CshB) [39]. Indeed, in that particular mutant, *mpfA* mRNA is overexpressed, leading to slow growth. Additionally, we showed that deletion of *mpfA* leads to a magnesium hypersensitivity [39]. Here we show that a range of suppressor mutations in CorA2, a CorA paralog, as well as overexpression of MgtE are equally able to compensate for overexpression of MpfA. Coupling these results with a wide range of deletion mutants and a second genetic screen for suppressors of *ΔmpfA* magnesium hypersensitivity enables us to propose a model of Mg^2+^ homeostasis in *S*. *aureus*, which relies on two independently organised systems of import and export.

## Results

### Missense mutations in CorA2 confer a gain-of-function phenotype similar to mpfA knock-out mutations

We recently described the identification of MpfA (Magnesium Protection Factor A), which is essential for growth of *S*. *aureus* in high Mg^2+^ concentrations [39]. We identified MpfA in a screen for mutants that suppress the slow growth phenotype of a *ΔcshB* mutant on the synthetic RPMI medium. CshB, is one of two DEAD-box RNA helicases of *S*. *aureus*. Its cellular function remains to be defined, but interestingly the *mpfA* mRNA is highly expressed in a *ΔcshB* strain, explaining why loss of MpfA is beneficial in this genetic background [39].

We speculated that identification of additional non-*mpfA* suppressor mutations of *ΔcshB* could help identifying additional factors needed to maintain correct intracellular Mg^2+^ levels. We therefore isolated additional suppressors of *ΔcshB*, and sequenced the full genome of four of them. Two of these four suppressors had missense mutations in the SA2166 gene, a paralog of *corA* which we therefore tentatively name *corA2*. Our previous suppressor screen [39] had yielded 15 suppressors of which 10 were *mpfA*-mutants, and we proceeded to amplify and Sanger-sequence the *corA2* locus of the five remaining mutants, which revealed an additional 3 *corA2* missense mutations (Table 1). Thus, of the 19 isolated suppressor mutants from the two studies, 10 were mutated in *mpfA* and 5 in *corA2*.

**Table 1:** Suppressor mutants harboring mutations in *corA2*.

Allele	Exact mutation on chromosome	Sequencing
CorA2 S237R	Single base substitution T to A at position 2408749	WGS
CorA2 M250I	Single base substitution G to T at position 2408788	WGS
CorA2 Y183N	Single base substitution T to A at position 2433358	SS
CorA2 A186T	Single base substitution G to A at position 2433367	SS
CorA2 T227I	Single base substitution C to T at position 2433491	SS

Five out of nineteen *ΔcshB* suppressors harbor a mutation in *corA2*. All mutations are missense. We either sequenced the full genome of the strain (WGS: Whole Genome Sequencing) by Illumina, or only the *corA2* locus by Sanger Sequencing a PCR product (SS). Positions refer to the *S*. *aureus* N315 genome.

All suppressors identified in CorA2 are missense mutations (Table 1). Although CorA proteins share little sequence similarity, their function and structures are highly conserved, as is evidenced by the very similar structure of CorAs from *Thermotoga maritima* (TmCorA) and *Methanocaldococcus janaschii* (MjCorA) (S1 Fig) and the trans-kingdom complementation of *corA* mutants [19]. As such, mapping the location of the changed amino acids onto the two different known crystal structures can give a general idea of the position of said mutations, even though the residues may be different (S1 Fig). All five mutations seem to be located in the cytosolic part of the protein and not affecting residues known to be essential to CorA function, although our results show that they clearly have an impact on the growth of the *ΔcshB* mutant (S2 Fig).

To confirm that the identified mutations in CorA2 are truly responsible for the observed *ΔcshB* suppressor phenotype, we re-constructed 4 of the 5 point mutations (A186T, T227I, S237R and M250I) in *ΔcshB* and wild type genetic backgrounds. It is to be noted that these alleles were remarkably hard to clone in *E*. *coli*, due to very slow growth of the transformants and the frequent occurrence of additional mutations in the clones we obtained (see Material and methods). All four reconstructed *corA2* mutant alleles were able to suppress the *ΔcshB* growth phenotype, confirming that a single missense mutation in *corA2* is sufficient to generate an effect similar to a full deletion of *mpfA*. In contrast, a deletion of *corA2* was unable to suppress the *ΔcshB* growth phenotype (S2 Fig). The combined facts that the mutated *corA2* alleles appear to be toxic in *E*. *coli*, that we obtained only missense mutations in the suppressor screen, and that a deletion of *corA2* does not show a suppressor phenotype, strongly suggest that the mutated *corA2* alleles confer a gain or a change of function phenotype.

### Either MgtE and CorA2 are required for magnesium import

It has previously been shown in *Salmonella* that when a bacterium relies on multiple import systems for Mg^2+^, deletion of a single system can result in little to no phenotype. However, multiple deletions will lead to a need for high magnesium concentrations in the medium [40]. In addition to the two CorAs, *S*. *aureus* encodes a MgtE-like (SA0867) Mg^2+^ importer. In *Bacillus subtilis*, MgtE is the main magnesium importer and its expression is controlled by a magnesium sensitive riboswitch [26,41]. In *S*. *aureus mgtE* (SA0867) is the fifth gene in an operon, suggesting very different regulation mechanisms (S4 Table). Bioinformatics analyses predict the operon to encode a putative GTP pyrophosphokinase, a probable inorganic polyphosphase/ATP-NAD kinase, a putative pseudouridine synthase and a Na+/H+ antiporter-like protein, so the link with magnesium remains obscure, although none of the genes in this operon have been studied experimentally.

**Fig 1:**
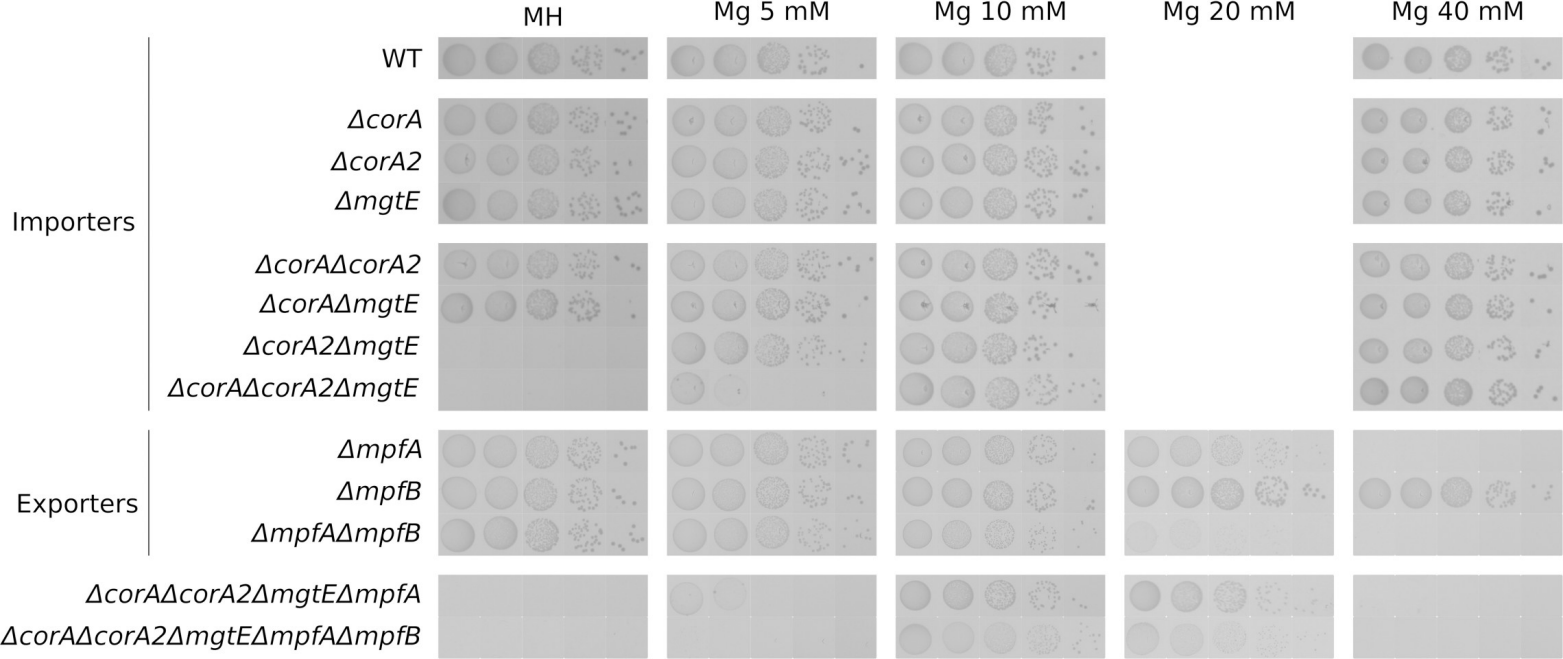
Import and export are two independent systems. Serial dilutions of overnight cultures of each strain were spotted on Mueller Hinton medium (MH) supplemented with increasing amount of MgCl_2_. Plates were incubated for 24h at 37°C.

**Fig 2:**
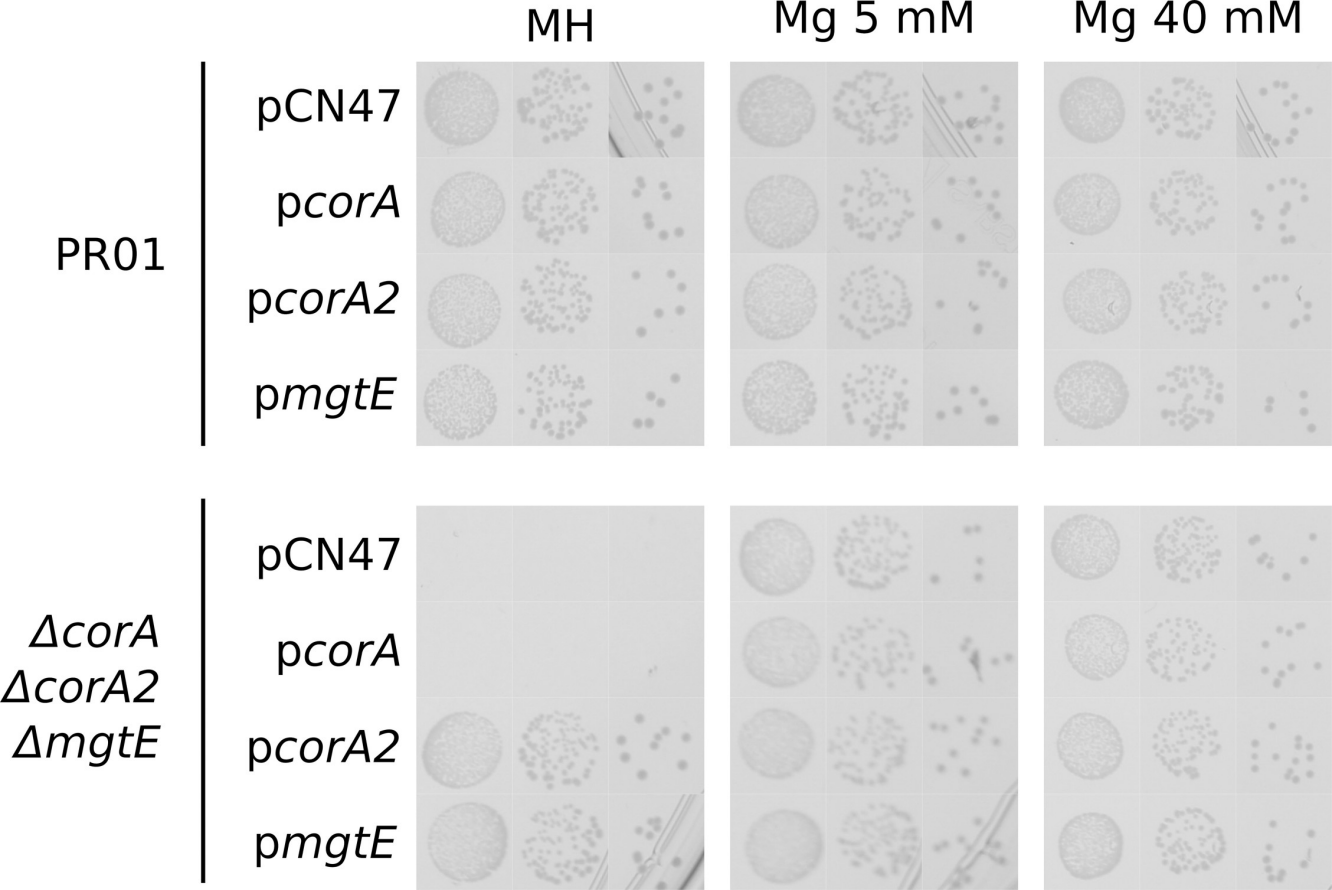
Complementation of *ΔmgtEΔcorAΔcorA2* by either pcorA2 or pmgtE *mgtE*, *corA* and *corA2* were cloned on pCN47 plasmids, a medium to high copy number plasmid (Charpentier et al., 2004). Since MgtE is in an operon, we cloned the ORF under the control of a constitutive promoter (Hu) while both CorAs were cloned with their natural promoter. Serial ten-fold dilutions of overnight cultures of each strain were spotted on Mueller Hinton medium (MH) supplemented with erythromycin and increasing amount of MgCl_2_. Plates were incubated for 24h at 37°C.

We were able to construct individual deletions of each of the genes putatively related to Mg^2+^ import: SA0867 (*mgtE*), SA2137 (*corA*) and SA2166 (*corA2*) and none of these single mutants display Mg^2+^ dependency (Fig 1, S3 and S4 Figs). However, while double deletion mutants *corA*/*corA2* and *mgtE*/*corA* grow as wild type, a double deletion mutant *mgtE*/*corA2* requires additional Mg^2+^ in the medium (5 mM added to the naturally occurring Mg^2+^ in MH medium) (Fig 1 and S4 Fig). A triple deletion mutant MgtE/CorA/CorA2 requires only slightly more magnesium to grow normally, indicating that CorA plays a minor role in Mg^2+^ import (Fig 1 and S4 Fig). The high magnesium dependency of this mutant can be removed by a plasmid expressing either *corA2* or *mgtE* but not *corA* (Fig 2). It is however important to note that the absence of a significant phenotype associated with *corA* could be imputed to an absence of expression in the tested conditions (S10 Fig). Magnesium import in *S*. *aureus* thus relies, at least under laboratory conditions, on either CorA2 or MgtE.

### MpfB (SA0780), an auxiliary magnesium export protein

The *S*. *aureus* genome encodes an MpfA paralog, SA0780 that we suggest to name MpfB. This protein shares a similar domain architecture to MpfA albeit with two main differences: the C-terminal extension is missing and the position equivalent to the glycine residue G326 of the sensing CBS domain, that we previously identified as essential to MpfA function, is replaced by an alanine in SA0780 (A301). Note that we previously reported that a SA0780 deletion caused slow growth on all medium types [39], but we have since discovered that this particular strain (PR01-59A) used in our previous study, unfortunately carries an additional point mutation in another apparently unrelated gene (SA1938^M252I^, pyrimidine nucleoside phosphorylase). A range of new *ΔSA0780* strains constructed for this study reveals no growth inhibition (S5 Fig) and we therefore assume that slow growth of PR01-59A is due to this SA1938^M252I^ mutation. A *ΔmpfB* strain is not magnesium sensitive and removing *mpfB* in a *ΔmpfA* background does not affect growth in absence of magnesium (Fig 1, S3 and S4 Figs). However, a *ΔmpfAΔmpfB* double mutant is slightly more sensitive to magnesium than *ΔmpfA* (Fig 1 and S4 Fig) suggesting MpfB does play a role in magnesium export, albeit minor.

### Import and export of magnesium appear to be two independent processes

The results presented here combined with previously published investigations on the function of MpfA [39] suggest a model where import of Mg^2+^ in *S*. *aureus* relies mostly on CorA2 and MgtE with an additional marginal role for CorA, while export of magnesium is carried out by MpfA.

Having identified factors for import as well as export of Mg^2+^, we wondered whether the two processes are coordinated, perhaps requiring common effectors, or they are two independent systems. This question is crucial for understanding Mg^2+^ homeostasis, since it has previously been reported that Mg^2+^ export in *Salmonella* via StCorC (a protein related to MpfA) was dependent on the presence of StCorA [8]. We constructed multiple deletion mutants of both the import and the export systems. These mutants grew neither without added Mg^2+^ nor at high Mg^2+^ concentrations, but only in a narrow ~10 mM Mg^2+^ range (Fig 1, S3 and S4 Figs). The presence of both low and high magnesium intolerance of the *penta* mutant strongly suggests that import and export function independently of each other.

### Mutations in *mpfA*, *corA2* and *mgtE* shift the homeostasis of free intracellular Mg^2+^

The changes to magnesium tolerance and requirements caused by the various mutations show that external Mg^2+^ concentrations have a dramatic effect on the growth of these mutants. But, does this mean that the internal Mg^2+^ homeostasis is affected, or are other factors at play?

The magnesium content of the bacterial cells is estimated to be in the hundred of millimolar range, but only a fraction of that quantity is “free”, i.e. not bound to membranes, proteins or nucleic acids [42]. It is therefore expected that methods measuring the total amount of metals in the cells cannot properly reflect changes in the free magnesium. Nonetheless, we used ICP-OES (Inductively Coupled Plasma Optical Emission Spectrometry) to quantify the total amount of magnesium of WT cells and different transporter mutants grown in presence of increasing amounts of extracellular magnesium (S6 Fig). The ICP-OES results show that the total amount of magnesium per cell is not influenced by the added extracellular amount of magnesium in either the WT or mutant strains. Moreover, this invariability extended to zinc and manganese, two other divalent cations which were examined simultaneously (S6 Fig).

Since total magnesium quantification did not deliver usable data, we decided to measure only the free intracellular Mg^2+^. To visualize free intracellular Mg^2+^ variations, we developed a reporter system based on a Mg^2+^ sensitive riboswitch. We constructed pMK-BSmgtE-GFP, a multi-copy plasmid, where the GFP gene is under the control of the *Bacillus subtilis mgtE* promoter including its riboswitch [41]. It is to be noted that *mgtE* genetic environment is different in *S*. *aureus*. Indeed, *mgtE* is the fifth gene in a six genes predicted operon and using published methodology we have not identified any M-box type riboswitch on the chromosome [43]. *In vitro* studies of the BSmgtE riboswitch have revealed its dynamic range to be between approximately 1 mM and 10 mM, with an Ec50 (concentration at which the activity is halfway between minimum and maximum) of 2.7 mM, and it is likely that the range is similar *in vivo* [41]. Outside this range, the riboswitch is either permanently off (>10 mM) or permanently on (<1 mM). The changes in free Mg^2+^ are therefore detected as an inverse function of the GFP fluorescence. The estimated internal concentration in bacterial cells is 1 mM, which falls on the lower end of the detection range, thus increases in internal magnesium concentration should be detectable with our reporter system, but probably not decreases [42].

We transformed the pMK-BSmgtE-GFP in some of our *S*. *aureus* strains including a wild type strain for reference, a mutant which does not grow in low magnesium *ΔmgtEΔcorA2*, a mutant which does not grow in high magnesium *ΔmpfA*, and *corA2*^*A186T*^. Each of the transformants was grown in a range of Mg^2+^ concentrations (from 0 to 40 mM Mg^2+^ added), (S7 Fig). The fluorescence of each culture was measured at mid-exponential phase. We verified that the fluorescence of a constitutive promoter (pHu) controlled GFP, is not correlated to the external magnesium concentration or strain-dependant and that therefore the measurement of GFP, under control of the riboswitch, reflected indeed the change in Mg^2+^ concentrations (S8 Fig). Although we observe some variations between strains and between external Mg concentrations, these only barely pass the threshold for statistical significance (p<0.05), and differ clearly from the differences we observe in with the Mg^2+^-sensitive riboswitch.

**Fig 3:**
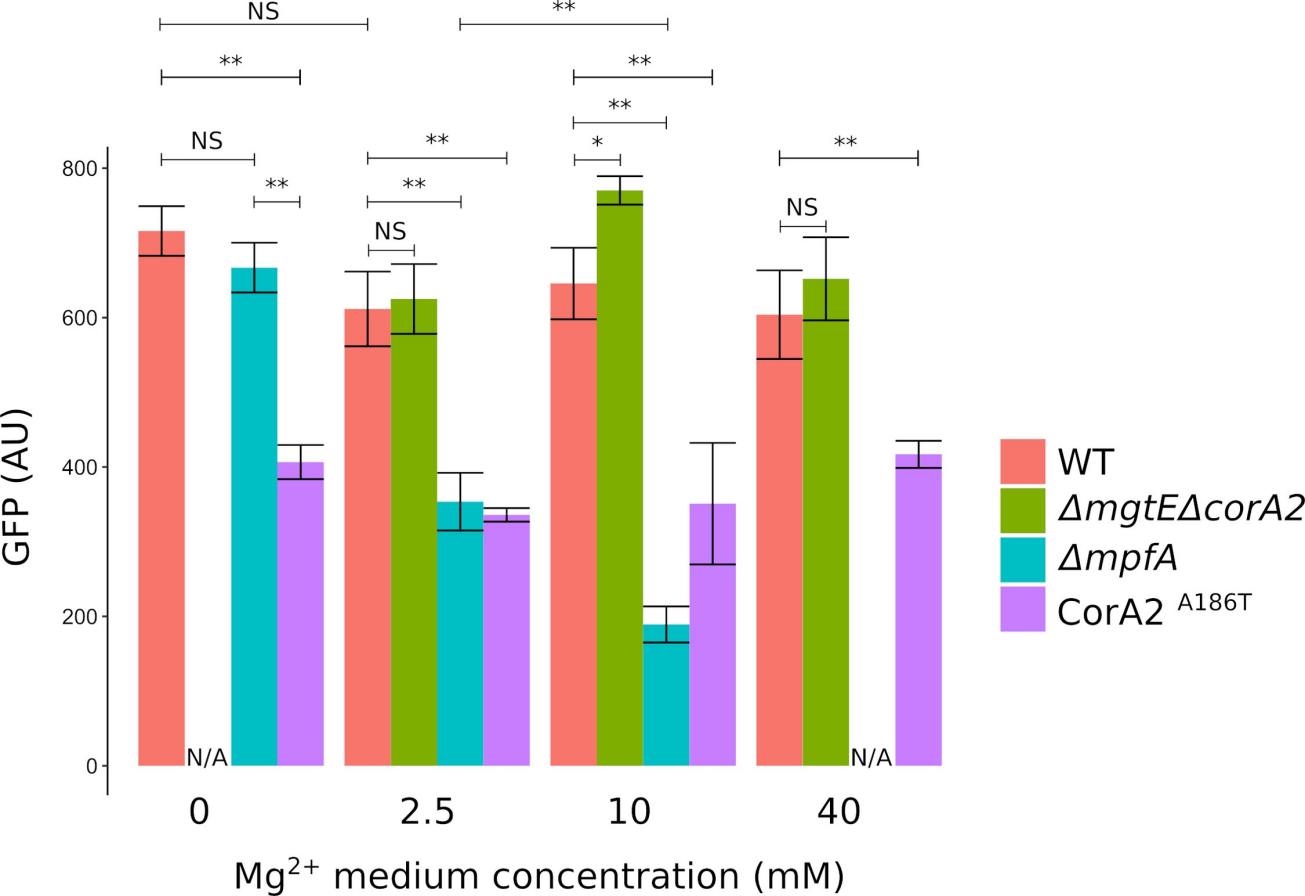
Internal free magnesium concentration is affected in magnesium transporter mutants. Fluorescence of cultures of different strains carrying a plasmid harboring a fusion between GFP and *BSmgtE* promoter where measured mid-exponential phase. Bacteria where grown in MH medium supplemented with indicated amount of MgCl_2_. The GFP fluorescence is given in arbitrary unit. The value was calculated as the average of three independent measurements (N = 3), subtracted of the background noise, i.e. the inherent fluorescence of a GFP-less culture. The results presented here are representative of at least three different experiments. N/A indicates the fluorescence could not be analysed since the strain does not grow in said condition. Unpaired t-test (R program) was used to calculate p-values. Numerical data are visible in S1 Appendix. * p-value<0.05 **p-value<0.01.

The signal from wild-type cells remained constant independent of the added magnesium, showing that *S*. *aureus* has efficient mechanisms to ensure Mg^2+^ homeostasis (Fig 3). The double *ΔmgtEΔcorA2* mutant shows fluorescence levels similar to, or slightly higher, than wild type. When grown in a medium supplemented with 2.5 mM Mg^2+^ this mutant grows significantly slower than wild type, suggesting internal Mg^2+^ is not sufficient for correct growth. Nevertheless, GFP fluorescence is as high as WT, presumably because the concentrations in both wild type and the double *ΔmgtEΔcorA2* mutant are below the sensitivity threshold of the riboswitch, and thus the fluorescence is already at its highest. The CorA2^A186T^ mutant systematically displays a significantly lower fluorescence than the wild type, indicating a high internal Mg^2+^ concentration. Interestingly this concentration is not affected by changes in external Mg^2+^ concentration, suggesting the mutant is still able to maintain homeostasis, albeit at a different level than the parental strain, consistent with *corA2*^A186T^ being a gain-of-function mutation. The *ΔmpfA* strain shows GFP levels similar to the wild type when no additional magnesium is present in the medium. The fluorescence drops to half of the wild type when 2.5 mM Mg^2+^ is added in the medium, a concentration that does not affect the growth of the strain (S6 Fig). At 10 mM supplemental magnesium the fluorescence drops to about 25% of the wild type, and growth is significantly slower. Thus, internal Mg^2+^ increases in *ΔmpfA* even at low levels of external Mg^2+^ and without affecting viability. Secondly, the upper limit sensitivity of the riboswitch is reached before the internal concentrations in Mg^2+^ increases above the growth inhibitory threshold.

### *ΔcshB*-suppressing mutations in *corA2* and *mpfA* act independently

**Fig 4:**
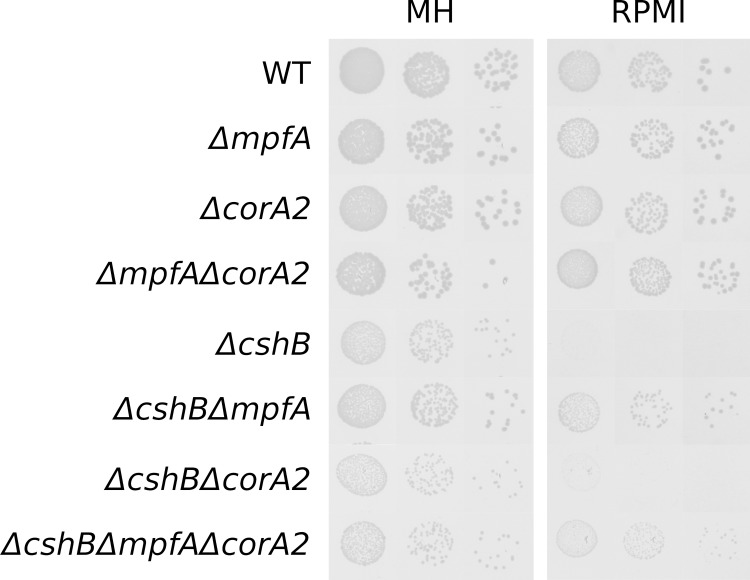
*mpfA* is epistatic to *corA2* in *ΔcshB* suppression. Three dilutions of overnight cultures of each strain were spotted on Mueller Hinton medium (MH) or RPMI medium supplemented with uracil. Plates were incubated for 24h at 37°.

We hypothesise that MpfA is an exporter but at present we cannot exclude that it acts as a regulator of export via *corA2*, as was suggested for the MpfA homolog StCorC [8]. If the latter were true, then the suppressor effect of a *mpfA* deletion in a *ΔcshB* strain should disappear in absence of CorA2. We therefore constructed a *ΔcshBΔcorA2ΔmpfA* strain (and a *ΔcorA2ΔmpfA* control), and observed that the triple deletion mutant grows slightly slower than *ΔcshBΔmpfA* but nonetheless retains the *ΔcshB*-suppressor phenotype (Fig 4) thereby excluding that MpfA acts via a modification of CorA2 transport activity.

Having established that *mpfA* is epistatic to *corA2* in *ΔcshB* suppression does not clarify whether the CorA2 point mutations influence the activity of MpfA. To probe this problem we tried to remove *mpfA* in strains carrying *corA2* mutated alleles (A186T and T227I) both in presence and in absence of *cshB*, using our selection/counter selection system [44]. Despite using this efficient technique we were however unable to delete *mpfA* in CorA2^A186T^ and CorA2^T227I^ backgrounds suggesting a synthetic effect of deleting *mpfA* in *corA2* gain-of-function background. Since our inability to get mutants is not proof of synthetic effect, we decided to measure the rate of loss of a temperature-sensitive *mpfA-*complementing plasmid in one the five CorA2 point mutants as an example. We first introduced a temperature-sensitive (TS) vector carrying the *mpfA* ORF under its promoter in a CorA2^A186T^ strain. In this strain we were able to easily replace *mpfA* with a chloramphenicol cassette. We then measured the loss of the complementation plasmid at non-permissive temperature, to determine whether *mpfA* is essential in a CorA2^A186T^ background. We verified that the temperature switch allowed loss of a *mpfA-less* temperature-sensitive backbone in all tested strains. A diluted overnight culture (~1000 CFU) grown at permissive temperature with antibiotic, was plated on rich medium without antibiotic at non-permissive temperature. A hundred colonies were then restreaked on rich medium with and without antibiotic to assess the rate of plasmid loss. The control plasmid was readily lost in all strains tested and as expected, the complementing plasmid is not required in the *corA2*^*A186T*^ strain (78% loss). However, the two independent *ΔmpfA corA2*^*A186T*^ cultures kept the *mpfA*-encoding plasmid significantly more often (only 2% and 5% loss respectively). The significantly lowered rate of plasmid loss suggests a synthetic enhancement. This rate seems too high to be due to additional spontaneous suppressor mutations and full genome sequencing of the seven isolated plasmid-free *ΔmpfA corA2*^*A186T*^ strains detected no additional mutations but confirmed the presence of the *corA2*^*A186T*^ and the deletion of *mfpA*. Thus, these two mutations are not synthetic lethal even though the strain was very difficult to construct. Additionally, a strain carrying these two mutations behaves as a *ΔmpfA* strain, being as sensitive to magnesium and still suppressing *ΔcshB* slow growth (S9 Fig).

### Magnesium homeostasis is disturbed in cshB mutants

**Fig 5:**
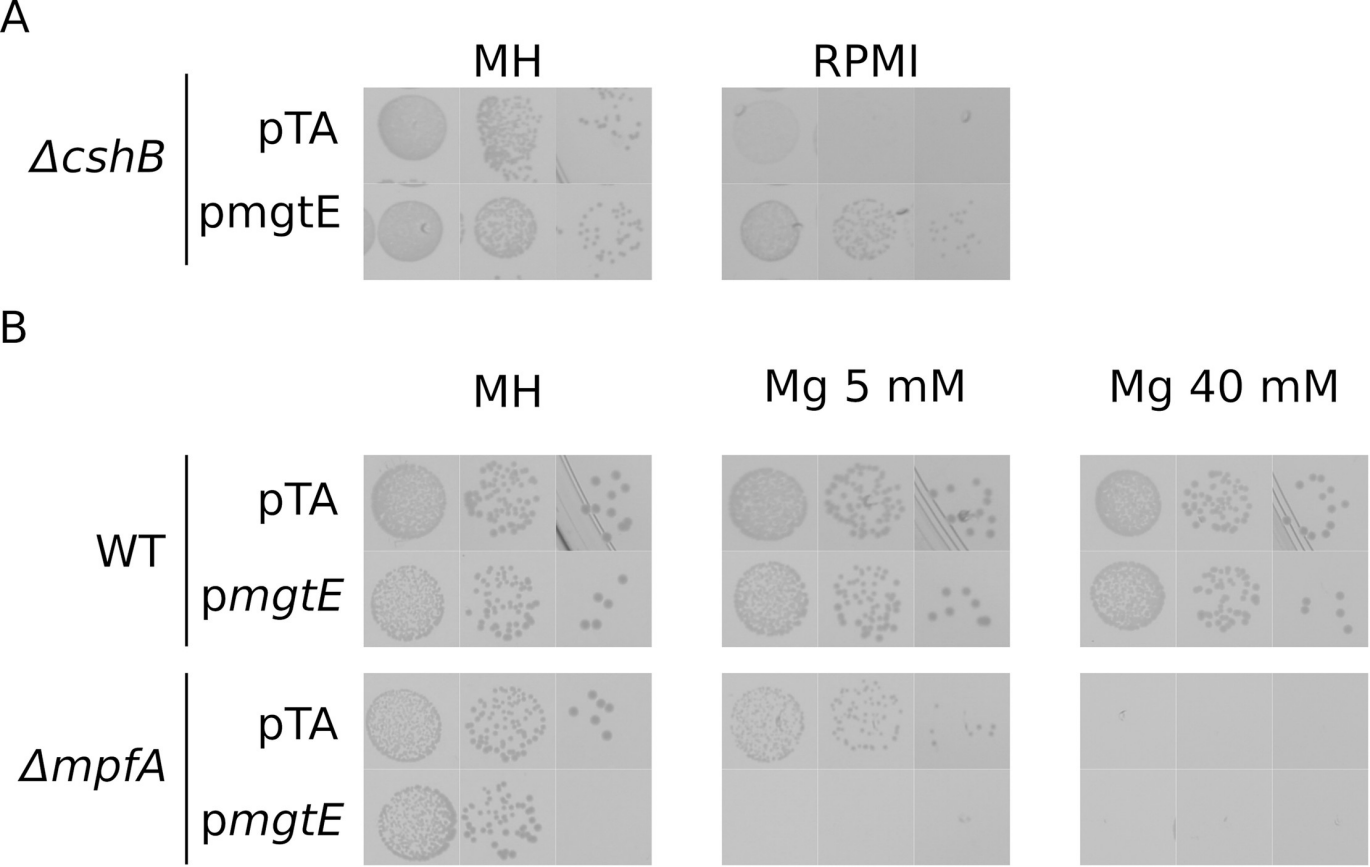
Overexpression of MgtE affect both *ΔcshB* and *ΔmpfA* mutants. *SamgtE* was clone under a constitutive promoter (pHU) on a pCN47 derived vector, carrying a tetracycline cassette. All plates were supplemented with tetracycline and incubated for 24h at 37°C. A: Three dilutions of overnight cultures of *ΔcshB* carrying either an empty plasmid or the fusion were spotted on Mueller Hinton medium (MH) or RPMI medium supplemented with uracil. B: Three dilutions of overnight cultures of WT and *ΔcshB* were spotted on Mueller Hinton (MH) plates supplemented with the indicated amount of MgCl_2_.

The present results show that both MpfA and CorA2 play key roles in maintaining magnesium homeostasis in *S*. *aureus*. Since the vast majority of suppressors of *ΔcshB* is mutated in either of these genes, *ΔcshB* phenotypes can be linked to a lowering in internal magnesium concentration caused by overexpression of MpfA. Such a change unfortunately falls below the dynamic range of the BSmgtE riboswitch reporter construct we developed. We can however predict that if this hypothesis is correct, an increase in magnesium import would compensate the leaking of magnesium and thus improve growth of *ΔcshB*, such as what is observed in CorA2 point mutants. Thus, the overexpression of MgtE from a multi-copy plasmid should also improve *ΔcshB* growth, which is exactly what we observed (Fig 5A). Conversely, such an increase should be deleterious to a *ΔmpfA* strain, incapable of exporting excess magnesium. Indeed overexpression of MgtE from a multi-copy plasmid increases *ΔmpfA* magnesium sensitivity (Fig 5B).

### Overexpression of MpfB or gain-of-function mutations in MpfB can compensate for the lack of MpfA

The magnesium hypersensitivity of a *ΔmpfA* mutant shows MpfA is a main player in magnesium export. However, when growing this mutant in presence of high magnesium, we repeatedly observed suppressor mutations. Hoping to get more insight into MpfA and magnesium homeostasis, we isolated and sequenced seven spontaneous suppressor mutants capable of growing in presence of 80 mM MgCl_2_ (Table 2). Most of these suppressors carry missense mutations in *mpfB*. Since we already showed that deletion of *mpfB* does not improve *ΔmpfA* magnesium sensitivity, these mutations are very probably gain-of-function. However, in absence of a structure of the protein or a close orthologs, we cannot make predictions as to how these mutations might improve MpfB function. Nevertheless, it is interesting to note that two of the mutations are located in the predicted transmembrane domain (T28I & E53K) and the other three in close proximity with each other in between the membrane domain and CBS sensing domain (S148F, L166I, G170R).

**Table 2:** Spontaneous mutations suppressing *ΔmpfA* magnesium sensitivity.

Genomic position*	Mutation	Gene	Protein change
892843	C to T	*mpfB* (SA0780)	T28I
892917	G to A	*mpfB* (SA0780)	E53K
893203	C to T	*mpfB* (SA0780)	S148F
893256	T to A	*mpfB* (SA0780)	L166I
893268	G to A	*mpfB* (SA0780)	G170R
1915575	T to C	tRNA38 (tRNA-fmet)	NA

* Positions refer to the *S*. *aureus* N315 genome.

**Fig 6:**
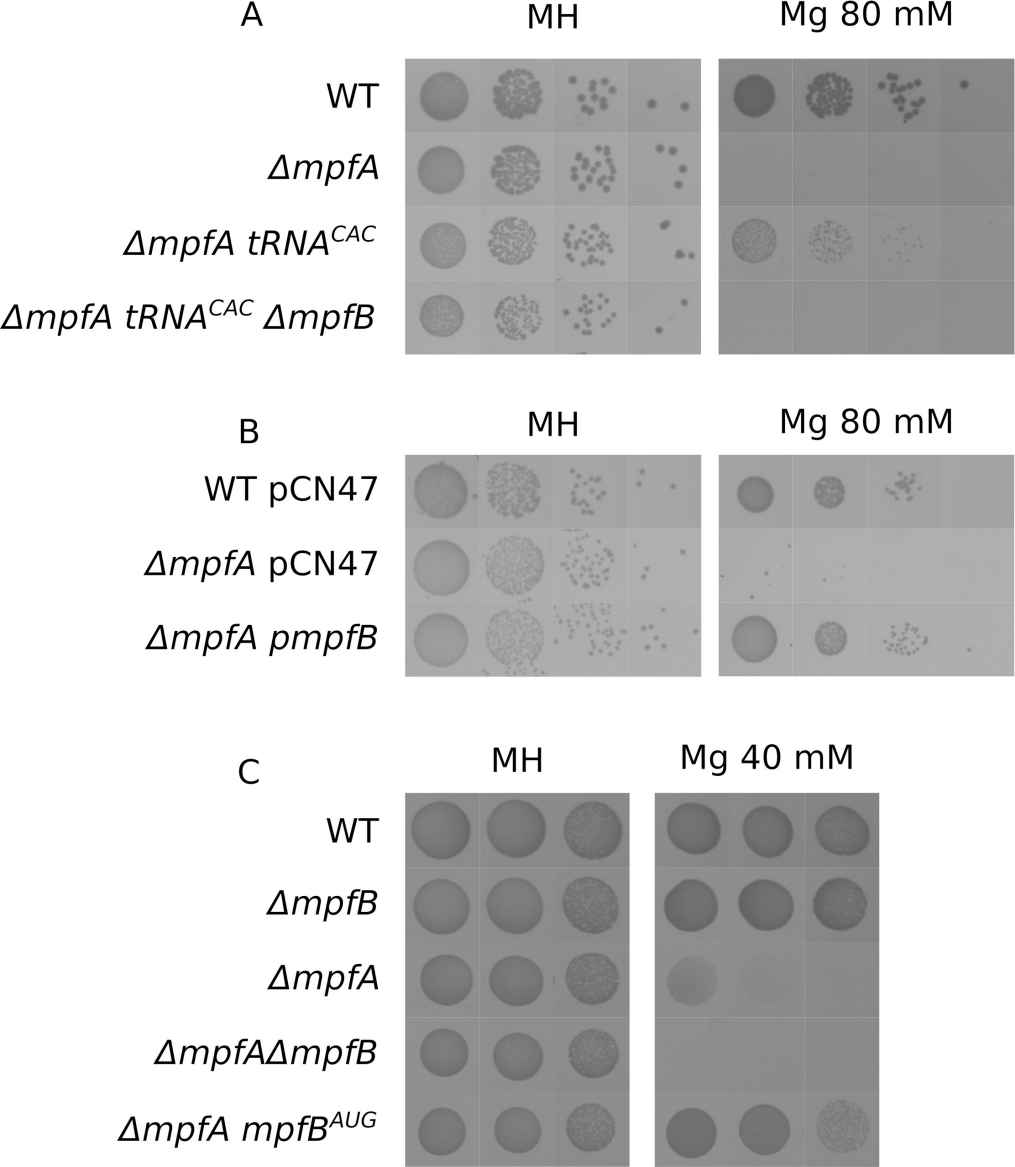
MpfB is an active protein. Serial dilutions of overnight cultures of each strain were spotted on Mueller Hinton medium (MH), supplemented in uracil and eventually supplemented with 40 or 80 mM MgCl_2_.Plates were incubated for 24h at 37°C. A: In absence of *mpfB*, tRNA^CAC^ mutation does not suppress *ΔmpfA* magnesium sensitivity. B: Expression of MpfB from a multi-copy plasmid (pCN47) complements *ΔmpfA* magnesium sensitivity. Plates were supplemented with erythromycin. C. An AUG start codon for *mpfB* suppresses *ΔmpfA* magnesium sensitivity.

The most surprising suppressor mutation arose in tRNA38, one the two initiator tRNAs of *S*. *aureus*. These tRNAs are responsible for initiation of translation, carrying a fMet (Formylmethionine) and recognizing the AUG codon. The mutation we obtained is a substitution changing the third residue of the anticodon from CAU to CAC, suggesting this mutant tRNA is better suited to initiate translation of GUG starting reading frames. These genes are significantly less well translated than AUG starting ones [45]. Thus we formulated the hypothesis that this tRNA mutation leads to the improved translation of one or several of GUG starting genes. *S*. *aureus* genome encodes at least 180 of them, amongst which is *mpfB*. If *mpfB* is indeed the gene whose improved translation relieves *ΔmpfA* magnesium hypersensitivity, we can make two predictions. 1/ Deletion of *mpfB* in the the tRNA38^CAC^ background should reverse the suppressor phenotype, i.e. the mutant would be magnesium sensitive, 2/ overexpression of *mpfB* should complement *ΔmpfA*. Indeed the tRNA38^CAC^ mutation is unable to suppress the magnesium hypersensitivity of ΔmpfA in absence of *mpfB* (Fig 6A). Moreover, introduction of *mpfB* on a multi-copy plasmid significantly improves *ΔmpfA* growth on high magnesium (Fig 6B). Finally, we decided to mimic the phenotype of tRNA38^CAC^ mutation by mutating the first codon of *mpfB* from GUG to AUG (Fig 6C). That allele also relieves *ΔmpfA* magnesium sensitivity strongly indicating that improved translation initiation of *mpfB* is enough to compensate the absence of *mpfA*.

## Discussion

Bacteria require significant amounts of magnesium to grow, since magnesium is the most abundant divalent cation in cells, reaching hundreds of millimolars. To acquire such high quantities of this metal ion bacteria possess efficient and generally well-studied import systems. However, we have previously shown that when Mg^2+^ is too abundant, *Staphylococcus aureus* relies on magnesium export to maintain proper magnesium balance. Pathogens such as *S*. *aureus* encounter such abundance of Mg^2+^ naturally since it colonizes kidneys and bones, which are both magnesium-rich niches. Importantly, such high magnesium in these organs can not only drive the appearance of strains of intermediate resistance to the antibiotic vancomycin (VISA: Vancomycin Intermediate *S*. *aureus*), but also explains the different patterns of infection based on the ability to support or not these concentrations [36,38]. These studies highlight the importance of magnesium and its homeostasis both in the life of the bacteria and during infection.

The importance of magnesium homeostasis is exemplified by the phenotypes of a *ΔcshB* mutant [39]. CshB is a DEAD-box RNA helicase, a family of proteins known to be involved in all stages of RNA regulation, transcription, translation and decay. In absence of CshB, *S*. *aureus* is cold sensitive and grows poorly on RPMI media, two phenotypes which are related to overexpression of *mpfA* [39]. Both the loss of *mpfA*, as well as gain-of-function mutations in CorA2, can restore the slow growth of *ΔcshB*. This strongly suggests a magnesium imbalance is at the origin of the slow growth of *ΔcshB*. Overexpression of another well-characterized Mg^2+^ importer, MgtE, also improves *ΔcshB* growth, further supporting this magnesium imbalance hypothesis. We are well aware that, although these genetic interactions and the known functions of the involved genes make this model enticing, hard data showing magnesium imbalance in *ΔcshB* are required to prove this point. Moreover, it is important to note the identified proteins, MgtE and CorA2 have not been properly characterized in *S*. *aureus* and thus their specificity was not determined. It remains possible that these proteins act on other ions. A proper biochemical study of these proteins and MpfA is necessary to ensure they are indeed magnesium transporters. Additionally, it is difficult to understand how the *ΔcshB* defect in magnesium homeostasis would be aggravated by growth in the cold or in RPMI. We speculate that this magnesium imbalance leads to growth defect of *ΔcshB* by affecting specific CshB targets, yet to be identified.

**Fig 7:**
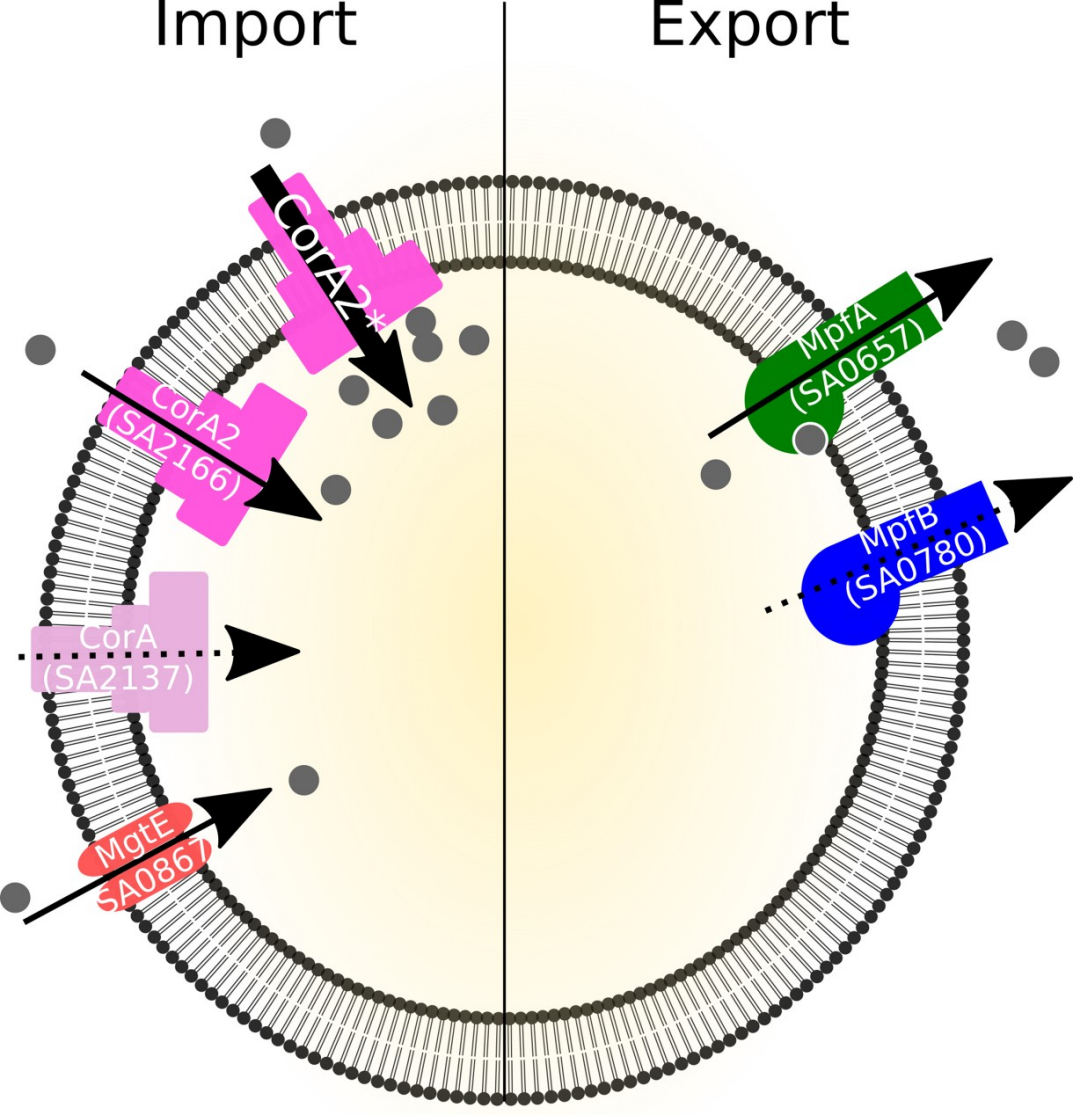
Current model of magnesium homeostasis in S. aureus. Data presented here allows us to propose the following model. Magnesium import is carried mainly by CorA2 and MgtE with additional marginal role of CorA. We have isolated mutated alleles of CorA2 which are able to increase magnesium import. Export is ensured by MpfA, although it remains to be determined whether MpfA directly transport Mg^2+^ or regulates another player. MpfB, a paralog of MpfA plays an additional minor role. Arrows indicate the proposed directionality of the transporter, full arrows to show the main transporters and dotted arrows for the secondary actors.

The use of the *ΔcshB* phenotype and genetic tools to probe Mg^2+^ imbalance, has allowed us to identify key players in magnesium homeostasis, both for import and export, in the pathogenic bacterium *S*. *aureus*. The import system is composed of two main proteins, CorA2 (SA2166) and MgtE (SA0867), and an accessory CorA (SA2137), while the export system is composed essentially of MpfA (SA0657) with a smaller role for MpfB (SA0780) (Fig 7). Furthermore, import and export constitute two independent systems as evidenced by the additivity of the phenotypes of the deletion mutants (Fig 1).

We have determined that, at least in laboratory conditions, *S*. *aureus* uses either MgtE or CorA2 to import magnesium, since only a double deletion mutant showed defect in growth without added magnesium. This result raises the question of the function of this redundancy. Multiple import systems are usually useful in different conditions. For example, in *Salmonella* MgtA acts as the active transport system in extremely low magnesium concentrations (<10μM) while CorA is the main transporter in physiological range of this metal ion. The conditions in which either CorA2 or MgtE are important need to be explored. In addition, *S*. *aureus* encodes two CorA alleles, with CorA2 being the main player in laboratory conditions. Interestingly, phylogeny shows that CorA2 is specific to firmicutes while CorA is more closely related to proteobacterial CorA proteins [12]. Finally, the question of the expression levels of these proteins needs to be further studied to properly explore the mechanisms of this redundancy.

Maintaining a proper balance of magnesium levels, especially in high magnesium conditions requires an export mechanism. Surprisingly, only two studies by the Maguire group have focused on magnesium export in bacteria. Both focused on the role of CorA in Mg^2+^ export in *Salmonella* [8,40]. The authors showed that in absence of CorA no export was observed. Moreover, export could also be abolished in a triple deletion mutant CorBCD, thus showing that Mg^2+^ export in *Salmonella* requires the presence of both CorA and at least one of CorB, CorC or CorD. We previously showed MpfA is essential to growth in high magnesium and, based on its similarity to both CorC and a eukaryotic family of magnesium transport mediators (CNNM), proposed it is the magnesium exporter of *S*. *aureus* [39]. While the deletion of *mpfB*, an *mpfA* paralog, does not yield an observable phenotype the double deletion mutant is even more Mg^2+^ sensitive showing that MpfB is functional. Strikingly, many spontaneous suppressor mutations in *mpfB* are capable of complementing *ΔmpfA*. We obtained many gain-of-function substitutions but also a mutant in an initiator tRNA that increases levels of MpfB. These results show that MpfB intrinsic activity is poorer than MpfA and can be improved but they also show that it is a functional protein and not a pseudogene. Unlike *mpfA*, *mpfB* is part of the *sigB*, the stress sigma factor, regulon, suggesting that MpfB might more important under stress conditions [46]. This is in accordance with recent findings in which high magnesium concentrations trigger the *sigB* response [38].

Although the genetic interactions presented here strongly point toward magnesium as the ion affected by the various mutants showed here, quantification of said ion would consolidate these results. In order to do so, we tried to quantify total magnesium amounts in the cell using ICP-OES (Inductively Coupled Plasma Optical Emission Spectrometry experiment). We could not identify changes in total magnesium that could be dependent on one of these transporters or the extracellular magnesium quantity. This observation, although underwhelming, is not very surprising since the “free” internal magnesium, which is the quantity affected by the transporters, accounts for a very small fraction of total magnesium. It is therefore, much more relevant, to measure this internal free magnesium. Currently available methods to estimate internal Mg^2+^ levels are either not specific enough (fluorescent dyes which also bind other cations) or not commercially available (^28^Mg isotope). We therefore developed a tool based on a fusion between a Mg^2+^ sensing riboswitch and *gfp*. This tool allowed us to show that loss of MpfA results in an increase in internal magnesium concentrations, fitting our hypothesis that MpfA drives magnesium export. Current evidences do not allow us to arbitrate between two hypotheses: either MpfA is a direct magnesium exporter or a regulator of magnesium export. This question is harder to settle than it seems, as evidenced by a current debate concerning the ability of members of the eukaryotic family CNNM to actually transport Mg^2+^ by themselves [31,32]. Data presented here, show that MpfA does not regulate any of the known Mg^2+^ transporters since *ΔmpfA* magnesium sensitivity remains in absence of CorA2 and MgtE, but we cannot exclude MpfA acts upon a currently unknown third party. Nevertheless, this is unlikely since we have not found a putative third party in our many genetic screens. Solving the structure of MpfA and electrophysiology experiments could help settle this. As evidenced by the riboswitch-GFP fusion, intracellular magnesium levels in *ΔmpfA* are significantly higher than wild type even when no growth phenotype can be observed (with 2.5 mM added Mg^2+^) (S6 and S7 Figs). Thus the window of tolerated internal magnesium concentration seems rather wide. This is further validated by the increase in internal Mg^2+^ of the CorA2 gain-of-function mutant, once again without effect on growth in laboratory conditions (S6 and S7 Figs).

In conclusion, relying on indirect evidence of magnesium transport through the use of a Mg^2+^-sensitive riboswitch, genetic interactions and magnesium sensitivity phenotypes, we have identified many if not all of the magnesium transporters in *S*. *aureus* and show that homeostasis relies on two independent import and export systems.

## Materials and methods

### Strains and media

Strains and plasmids used in this study are described in S1 and S2 Tables. Primers are described in S3 Table.

*Escherichia coli* DH5𝛼 strain was cultivated in LB medium at 37°C supplemented with 100 mg/l ampicillin if necessary.

*Staphylococcus aureus* strains were grown at 37°C, eventually 25°C when indicated, in Mueller-Hinton (MH) broth (BD) supplemented with 10 mg/l uracil and if necessary with 10 mg/l tetracycline, erythromycin or chloramphenicol. All liquid cultures were made under continuous agitation.

On plates, *S*. *aureus* cells were grown on Mueller-Hinton (MH 211443, BD Biosciences, Allschwil, Switzerland) broth supplemented with 10 mg/l uracil and with 10 mg/l tetracycline, erythromycin, chloramphenicol, 200 mg/l of 5-fluoroorotic acid (5-FOA: US Biological, Swampscott, MA, USA) or varying concentrations of MgCl_2_ (Sigma-Aldrich M2670), as necessary. Agar plates contained 13 g/l of agar (Agar bacteriology grade, PanReac AppliChem). RPMI plates contained RPMI-1640 buffered with HEPES (Sigma-Aldrich R7388) supplemented with 10 mg/l uracil.

Construction of mutants was performed by allelic replacement as previously described, using the pyrEF/5-FOA counter selection system [44]. Briefly, the method for making allelic exchanges on the chromosome in *S*. *aureus* involves cloning the mutated allele with its surrounding sequence (to allow homologous recombination) on a vector that replicates in *E*. *coli*, but is non-replicative in *S*. *aureus*. Once the construct is transformed into *S*. *aureus*, then two successive homologous recombination events will generate the exact mutated allele, without genomic scarring. During these constructions steps, strains are grown on Mueller Hinton medium, eventually supplemented with 5-FOA to select for the strains losing the vector. The (in)ability to grow on RPMI medium (i.e. a uracil-less medium) is monitored after introduction of the vector on the chromosome and after its loss, but the mutant strains are not grown on this medium.

We had great difficulties cloning the various mutated alleles of *corA2* in *E*. *coli*. All four alleles appeared to be toxic to *E*. *coli* to various degrees, while a wild type allele poses no problem. The allele with the M250I mutation proved to be impossible to obtain directly, since all *E*. *coli* transformants carried vectors with additional mutations in the *corA2* gene of their inserts, as verified by Sanger Sequencing of the vectors. We nevertheless decided to re-generate the M250I mutant, by using one of these double-mutated *corA2* constructs to generate the *S*. *aureus* strain SA2166^P32T/M250I^, whereupon we repaired the P32T mutation to obtain the CorA2^M250I^ allele. The two mutations in the P32T/M250I double mutant are sufficiently far from each other to allow a subsequent repair of the P32T mutation, again with allelic replacement, without having to clone the entire mutated *corA2* gene in the *E*. *coli* vector.

The mutated loci and the totality of the two homologous recombination region of all the strains constructed were sequenced by Sanger Sequencing of a PCR product. This was done to confirm the proper sequence of the intended mutation, whether a SNP or a deletion, and the absence of additional unwanted mutations. In the case of multiple mutants, the previously introduced mutations were verified Sanger Sequencing for SNP mutations and by PCR for deletions. SA0657 and SA2166 ORFs were replaced by an antibiotic resistance cassette (chloramphenicol and erythromycin respectively) while SA0780, SA2137 and SA0867 ORFs were deleted in frame. In particular, in the case of SA0867 the first 9 and last 17 codons were conserved to avoid a polar effect due to the deletion.

### Suppressor screens

*ΔcshB* strains carrying suppressor mutations were obtained by plating 10 μL of O/N culture (grown in Mueller Hinton) on RPMI plates supplemented with uracil. After 24h at 37°C, *ΔcshB* grows poorly allowing us to isolate colonies slightly bigger than background. One to two colonies per plate were selected. Selected colonies were further isolated at least two times on RPMI plates and their growth phenotypes on Mueller Hinton both at 37°C at 25°C and RPMI at 37°C were compared to the parental strain and WT strain. Only isolates showing improved growth compared to parental strain were further sequenced.

*ΔmpfA* strains carrying suppressor mutations allowing growth in presence of 80 mM MgCl_2_ were obtained in a similar fashion.

### Sequencing and bioinformatics

Whole genome sequencing was performed at the iGE3 Unige Genomics platform. Sanger sequencing was performed by Fasteris (Fasteris SA, Switzerland). Multiple alignments were performed using Clustal Ω (http://www.ebi.ac.uk/Tools/msa/clustalo/).

### Measurement of the concentration of free intracellular Mg^2+^

The BSmgtE riboswitch sequence was amplified from *Bacillus subtilis* (BS168) with oligos BSMgtERS_sph_F (GGGCATGCTGTTCCGTAATTGTGATGTAAG) and BSMgtERS_kpn_R (GGGGTACCCGGGACTCGTACCTCCTCTAC) and cloned on pMK4 carrying *gfp* [47].

200 μl of medium in 96 well plates were seeded at 1/100^th^ dilution with bacterial cultures and incubated at 37°C. Measurements of OD at 600 nm and GFP (485 nm/528 nm) were performed every hour. Only fluorescence levels at OD≥0.2 (mid-exponential phase) were considered. Autofluorescence of GFP-less cultures was measured on the same plate and subtracted from all values. Measurement were performed on a Synergy H1 plate reader (Bio-tek).

## Supporting information

S1 Table Strains used in this study. (XLSX)

S2 Table Plasmids used in this study. (XLSX)

S3 Table Primers used in this study. (XLSX)

S4 Table Genetic environment of magnesium homeostasis genes. (XLSX)

S1 Fig Mapping the mutated residues of CorA2 onto known CorA structures. A: Mutated residues in CorA2 and the corresponding residues in *Thermotoga maritima* (TmCorA) and *Methanocaldococcus jannaschii* (MjCorA) CorAs as defined by the panel D alignment. The mutations are color-coded for ease of reading and the colors are conserved across all panels. B: Table of percentage of identity between the CorA proteins as computed by ClustalΩ. C: The structure of CorA2 from *S*. *aureus* has not been resolved, the mutations are mapped onto homologous structures (TmCorA: pdb4i0u and MjCorA: pdb4ev6). The approximate position of the membrane is indicated. All five mutations seem to be located in the cytosolic part of the protein, with the M250 predicted to be located shortly before the transmembrane domain, in a part where the pore of CorA quickly widens. T227 and S237 are predicted to be part of the stalk helix, i.e. the part of CorA that initiates the movement that leads to the opening of the pore. D: Alignment of the sequences of *corA2*, *TmcorA* and *MjcorA*. Alignment performed using ClustalΩ (https://www.ebi.ac.uk/Tools/msa/clustalo/). Transmembrane domains are in light grey. The highly conserved GMN motif is boxed in red.
(PNG)

S2 Fig Point mutations in CorA2 can relieve *ΔcshB* RH slow growth and cold sensitivity. Serial dilutions of overnight cultures of each strain were spotted on Mueller Hinton medium (MH) or RPMI medium supplemented with uracil. Plates were incubated for 24 h at 37°C or 62 h at 25°C.
(PNG)

S3 Fig CFU quantification of various strains grown in presence of increasing concentrations of magnesium. A: Overnight cultures were washed twice in PBS and serially diluted (tenfold dilution at each step) 100**μ**L of solution was plated on MH agar plates containing indicated amounts of MgCl_2_. Dilutions 5, 6 and 7 were plated for conditions where a high CFU was expected, while dilutions 0, 1 and 2 were plated for conditions where a low CFU was expected. Two dilutions were counted for each condition and performed in biological triplicates. Calculation of mean and standard deviation were performed using the aggregate package in R. A star indicates conditions where suppressor mutants (confirmed by restreaking) arose on plates. B: Crop out of two of the plates used to count CFUs. A *ΔmpfA* strain grown on MH in absence of additional magnesium gives rise to colonies homogenous in size, at a high dilution (10^6^) while the same culture grown in presence of 40 mM magnesium gives rise to a heterogeneous colony population of suppressor mutants. The colonies observed are indeed suppressors as we confirmed by restreaking. C: Restreaking of 20 colonies from plates shown in panel B. *ΔmpfA* strain restreaked from plates without additional magnesium (bottom part) do not grow in presence of 40 mM magnesium unlike the spontaneous suppressors reastreaked from a 40 mM magnesium plate (top part).
(PNG)

S4 Fig Growth curves of magnesium transporter mutants. The indicated strains were seeded with 1/100^th^ of overnight grown cultures in 200**μ**L Mueller Hinton medium (MH) supplemented with uracil and the specified amount of MgCl_2_ in 96 well plates under continuous agitation at 37°C. OD600 was measured every half hour with an Epoch2 plate reader (Biotek).
(PNG)

S5 Fig Comparison of different ΔSA0780 (mpfB) mutants. Dilutions of overnight cultures of each strain were spotted on Mueller Hinton medium or RPMI medium supplemented with uracil. Plates were incubated for 21h at 37°C. Three, independently obtained *ΔSA0780* (*mpfB*) mutants were tested. PR01-59A, a *ΔSA0780* strain described in our previous study carries an additional mutation.
(PNG)

S6 Fig Quantification of metal ions by ICP-OES. Total amounts of magnesium, manganese and zinc were quantified by ICP-OES (Inductively Coupled Plasma Optical Emission Spectrometry experiment) adapted from a previously described protocol (Arabet et al, 2014). Briefly, 10^9^ bacterial cells of an exponential phase culture were harvested and lysed 10 min at 37°C with 1 mg/mL lysostaphin in 500**μ**L PBS. The samples were wet washed with 32.5% nitric acid (Suprapur, Merck) for 12 h at 100°C (Neumann et al. 2009) and were then filled to a tenfold volume with water prior to inductively coupled plasma optical emission spectrometry (ICP-OES) analysis. Two replicates for each sample were carried out, and the average concentration values were calculated. Analysis was performed using a ThermoFisher ICAP 6000 ICP-OES. A multielement standard solution (Merck) was used as a reference. Bacterial cultures were grown in Mueller Hinton medium supplemented with the indicated amount of MgCl_2_. The conditions are identical to that of Fig 3. N/A indicates the concentration could not be determined since the strain does not grow in said condition. Calculation of mean and standard deviation were performed using the aggregate package in R. Arabet D, Tempel S, Fons M, Denis Y, Jourlin-Castelli C, Armitano J, et al. Effects of a sulfonylurea herbicide on the soil bacterial community. Environ Sci Pollut Res Int. 2014;21: 5619–5627. doi:10.1007/s11356-014-2512-9
(PNG)

S7 Fig Growth curves of cultures used to measure GFP fluorescence. Bacteria where inoculated from overnight cultures at 1/100^th^ and grown in a 96 well plate at 37°C under continuous agitation in Mueller Hinton medium (MH). OD600 was measured every hour. Growth of bacteria (OD) carrying the plasmid harboring the BSmgtE-GFP fusion (BSMgtE) or the constitutive promoter-GFP fusion (Ctrl) is plotted as a function of time. The BSmgtE and Ctrl samples where grown on the same day, in the same plate.
(PNG)

S8 Fig Fluorescence of GFP under a constitutive promoter. Fluorescence of cultures of different strains carrying a plasmid harboring a fusion between GFP and a constitutively expressed promoter (pHU) where measured mid-exponential phase. Bacteria where grown in MH medium supplemented with indicated amount of MgCl_2_. The value was calculated as the average of three independent measurements (N = 3), subtracted of the background noise, i.e. the inherent fluorescence of the medium. The results presented here are representative of at least three different experiments. The BSmgtE and Ctrl samples where grown on the same day, in the same plate. The significantly different results (* p-value<0.05) are shown. Although we observe some variations between strains and between external Mg concentrations, these only barely pass the threshold for statistical significance (p<0.05), and are far removed from the clear differences we observe in Fig 3. Unpaired t-test (R program) was used to calculate p-values.
(PNG)

S9 Fig Double mutant CorA2^A186T^ ΔmpfA. A: Dilutions of overnight cultures of each strain were spotted on Mueller Hinton medium (MH) supplemented with uracil and eventually the indicated amount of MgCl_2_. Plates were incubated for 24h at 37°C. B: Dilutions of overnight cultures of each strain were spotted on Mueller Hinton medium (MH) or RPMI medium supplemented with uracil. Plates were incubated for 24h at 37°C.
(PNG)

S10 Fig RNA steady state levels of magnesium homeostasis genes. The RNA levels of the five genes implicated in magnesium homeostasis were measured by qRT-PCR. A: Graphical display of the cycle thresholds at which the different mRNAs were detected in a WT strain grown in absence of additional magnesium in MH medium at 37°C. All five RNAs can be detected albeit at varying levels. B: Comparison of expression of different targets between WT and *ΔcshB* strains, fold change compared to WT are plotted on the Y axis. C: qRT-PCR primers used in these experiments.
(PNG)

S1 Appendix Numerical data underlying Figs 3, S3, S4, S6, S7, S8 and S10. (XLSX)
