# Supplementary figures and images for: Genetic screens reveal novel major and minor players in magnesium homeostasis of Staphylococcus aureus
Source: PLoS Genet. 2019 Aug 15;15(8):e1008336. doi: 10.1371/journal.pgen.1008336 (PMC6711546; doi:10.1371/journal.pgen.1008336)

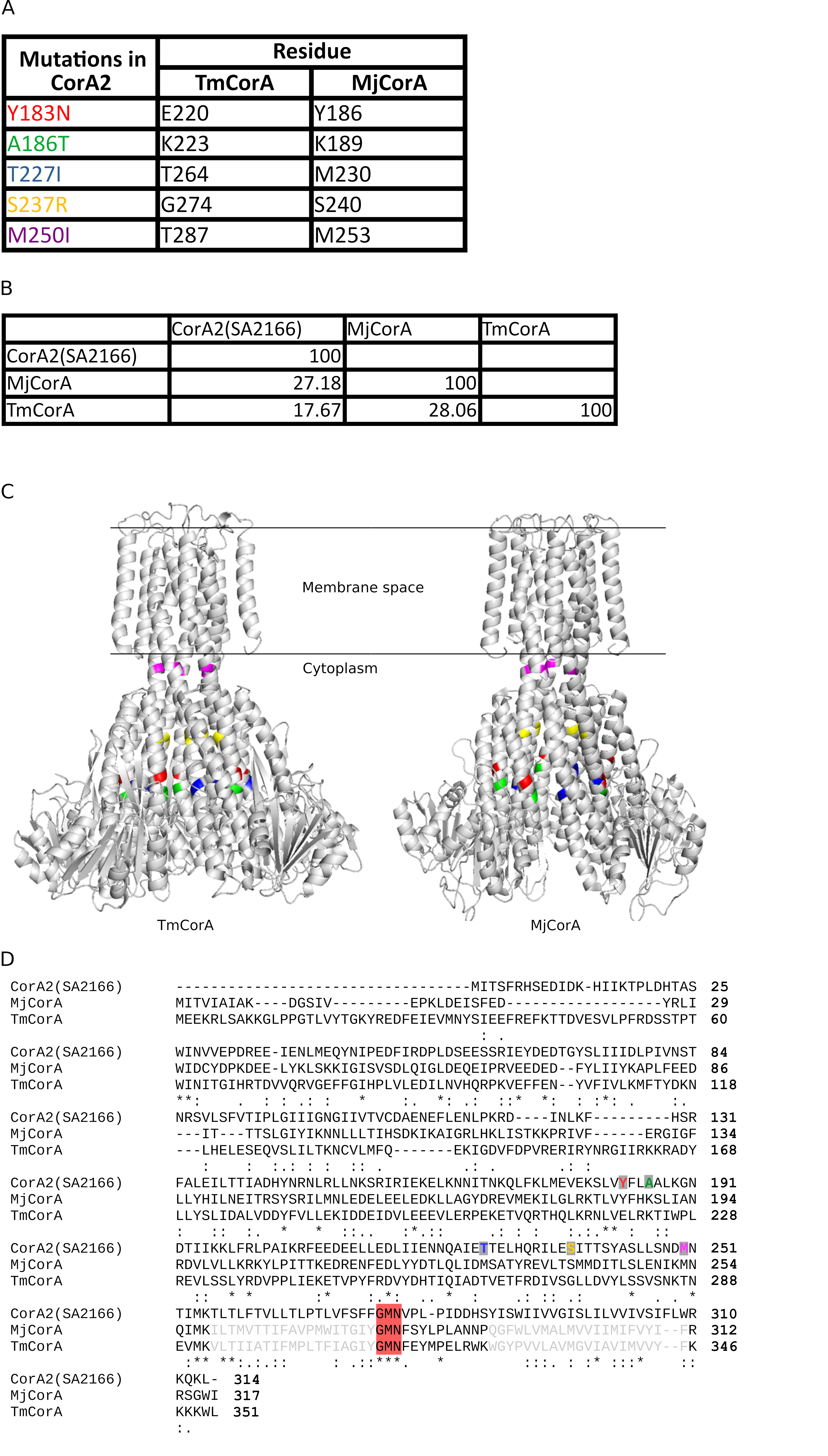

Supplement: S1 Fig — A: Mutated residues in CorA2 and the corresponding residues in Thermotoga maritima (TmCorA) and Methanocaldococcus jannaschii (MjCorA) CorAs as defined by the panel D alignment. The mutations are color-coded for ease of reading and the colors are conserved across all panels. B: Table of percentage of identity between the CorA proteins as computed by ClustalΩ. C: The structure of CorA2 from S. aureus has not been resolved, the mutations are mapped onto homologous structures (TmCorA: pdb4i0u and MjCorA: pdb4ev6). The approximate position of the membrane is indicated. All five mutations seem to be located in the cytosolic part of the protein, with the M250 predicted to be located shortly before the transmembrane domain, in a part where the pore of CorA quickly widens. T227 and S237 are predicted to be part of the stalk helix, i.e. the part of CorA that initiates the movement that leads to the opening of the pore. D: Alignment of the sequences of corA2, TmcorA and MjcorA. Alignment performed using ClustalΩ (https://www.ebi.ac.uk/Tools/msa/clustalo/). Transmembrane domains are in light grey. The highly conserved GMN motif is boxed in red. (PNG) [file pgen.1008336.s005.png]

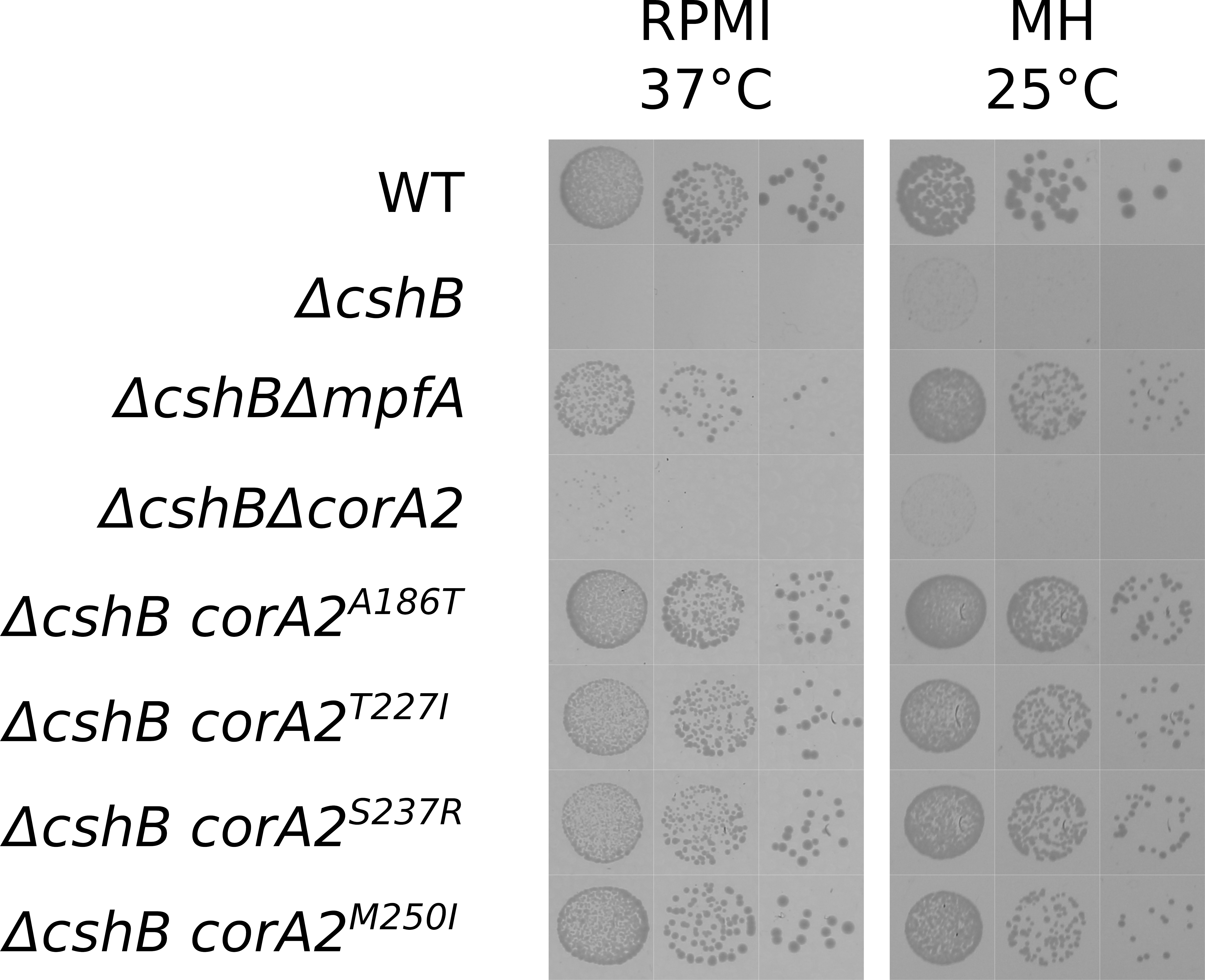

Supplement: S2 Fig — Serial dilutions of overnight cultures of each strain were spotted on Mueller Hinton medium (MH) or RPMI medium supplemented with uracil. Plates were incubated for 24 h at 37°C or 62 h at 25°C. (PNG) [file pgen.1008336.s006.png]

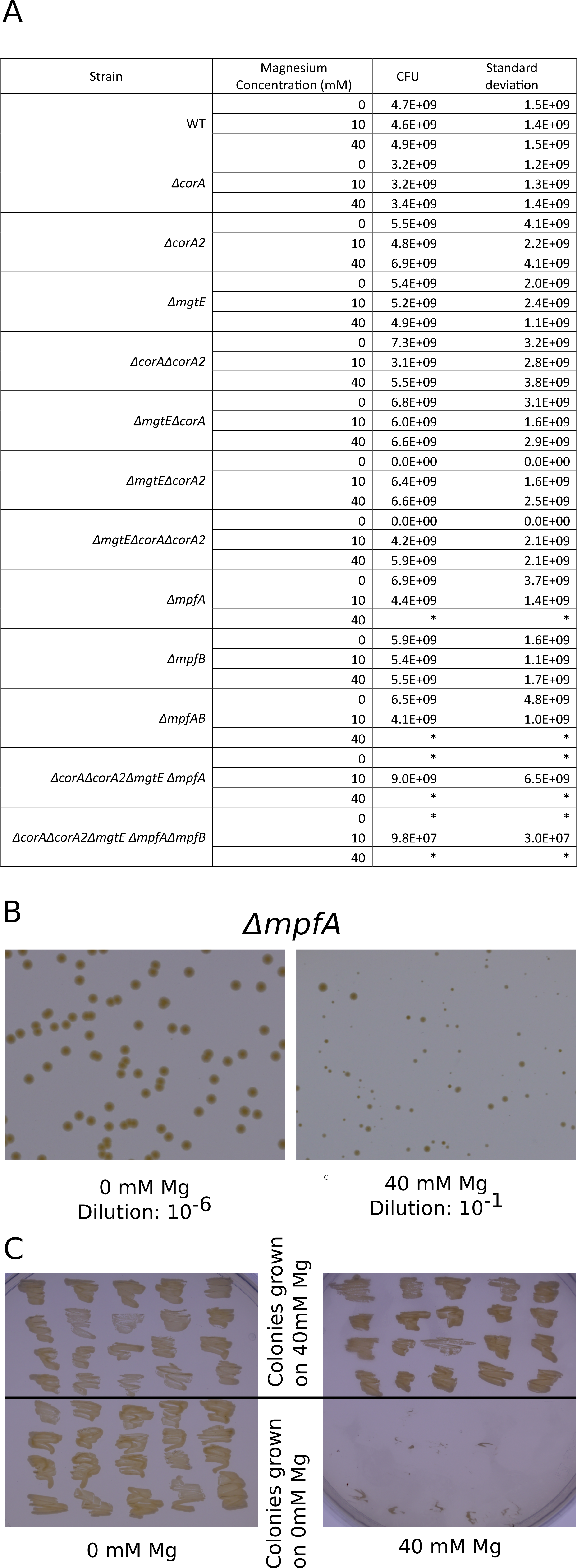

Supplement: S3 Fig — A: Overnight cultures were washed twice in PBS and serially diluted (tenfold dilution at each step) 100μL of solution was plated on MH agar plates containing indicated amounts of MgCl2. Dilutions 5, 6 and 7 were plated for conditions where a high CFU was expected, while dilutions 0, 1 and 2 were plated for conditions where a low CFU was expected. Two dilutions were counted for each condition and performed in biological triplicates. Calculation of mean and standard deviation were performed using the aggregate package in R. A star indicates conditions where suppressor mutants (confirmed by restreaking) arose on plates. B: Crop out of two of the plates used to count CFUs. A ΔmpfA strain grown on MH in absence of additional magnesium gives rise to colonies homogenous in size, at a high dilution (106) while the same culture grown in presence of 40 mM magnesium gives rise to a heterogeneous colony population of suppressor mutants. The colonies observed are indeed suppressors as we confirmed by restreaking. C: Restreaking of 20 colonies from plates shown in panel B. ΔmpfA strain restreaked from plates without additional magnesium (bottom part) do not grow in presence of 40 mM magnesium unlike the spontaneous suppressors reastreaked from a 40 mM magnesium plate (top part). (PNG) [file pgen.1008336.s007.png]

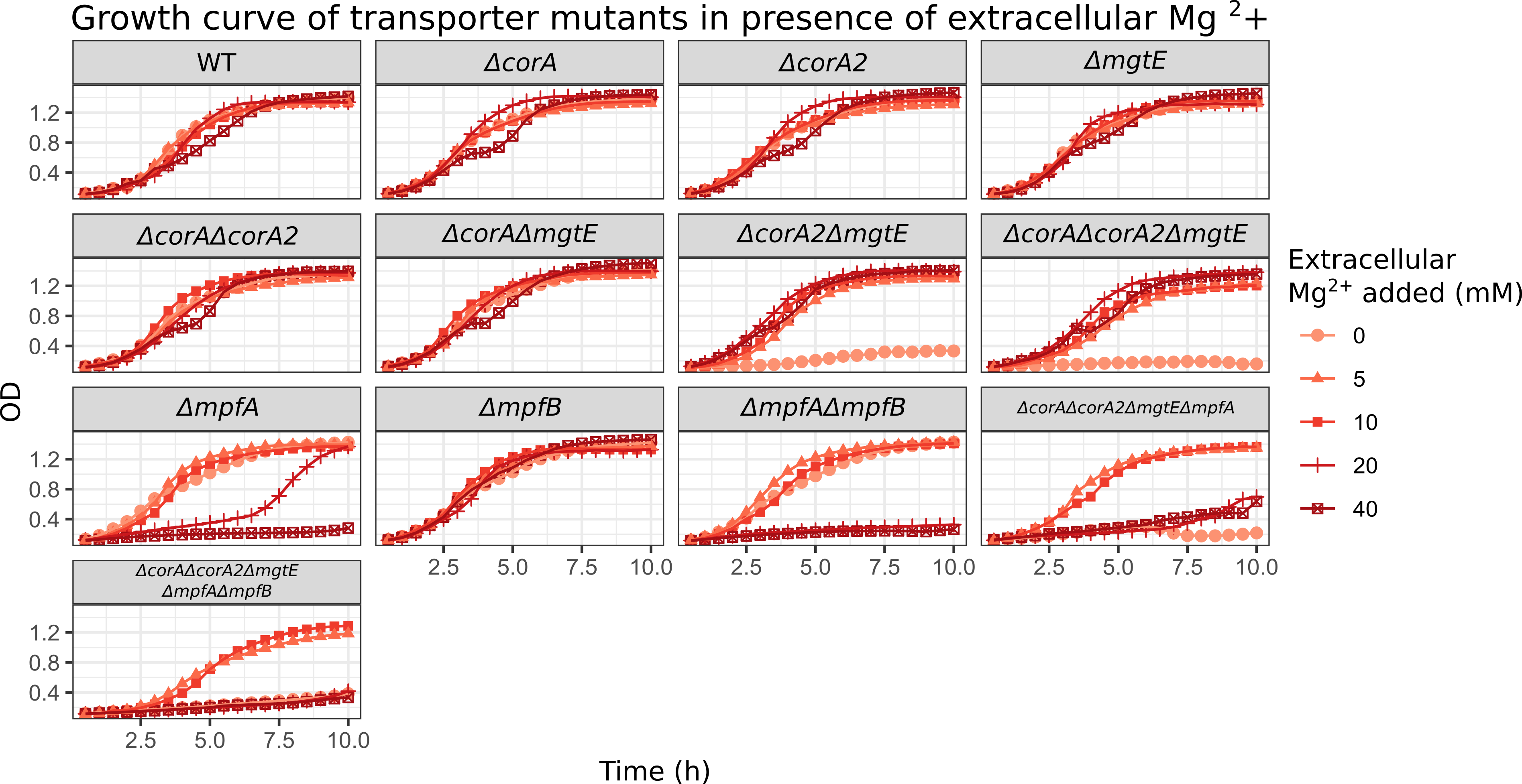

Supplement: S4 Fig — The indicated strains were seeded with 1/100th of overnight grown cultures in 200μL Mueller Hinton medium (MH) supplemented with uracil and the specified amount of MgCl2 in 96 well plates under continuous agitation at 37°C. OD600 was measured every half hour with an Epoch2 plate reader (Biotek). (PNG) [file pgen.1008336.s008.png]

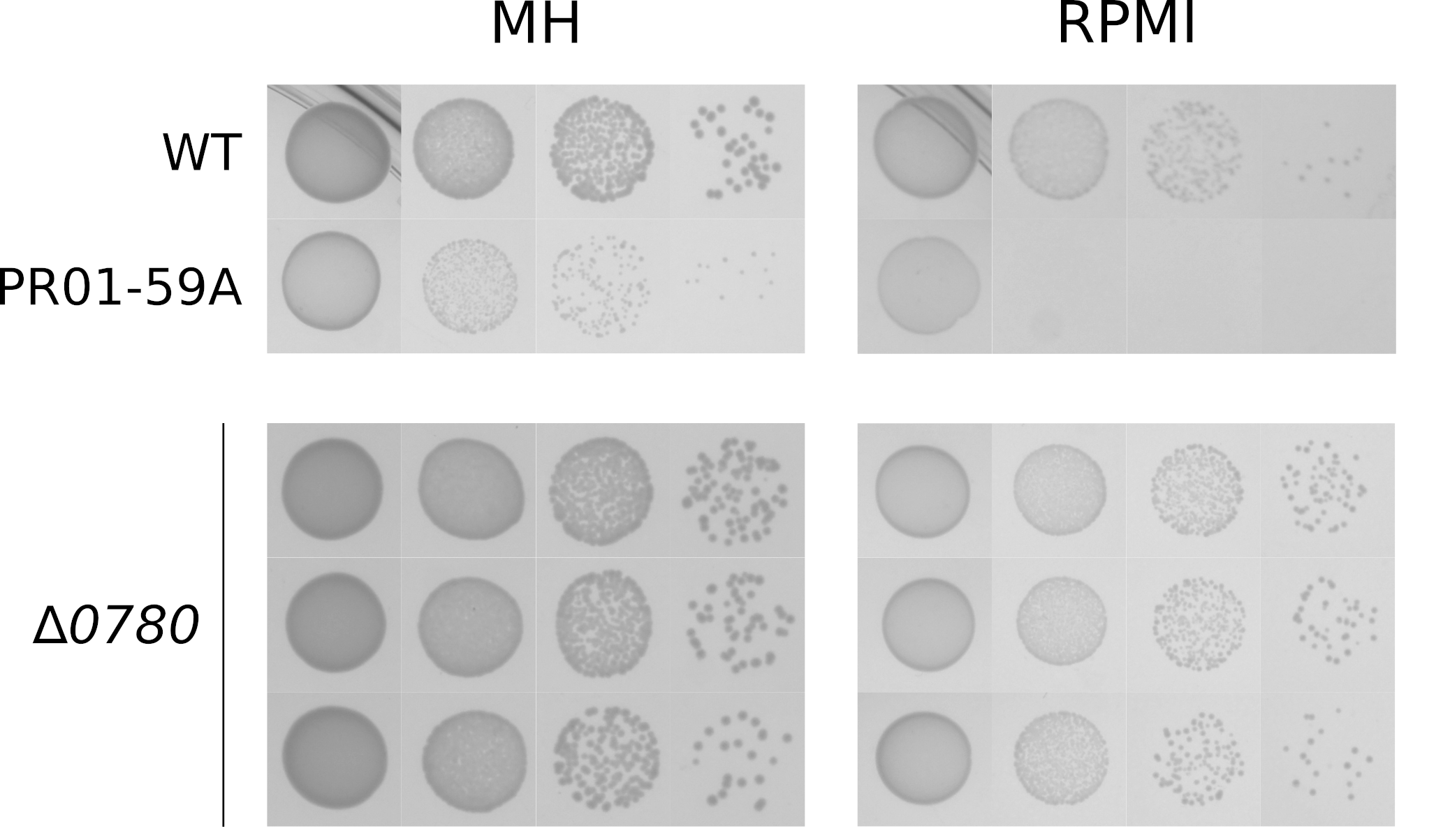

Supplement: S5 Fig — Dilutions of overnight cultures of each strain were spotted on Mueller Hinton medium or RPMI medium supplemented with uracil. Plates were incubated for 21h at 37°C. Three, independently obtained ΔSA0780 (mpfB) mutants were tested. PR01-59A, a ΔSA0780 strain described in our previous study carries an additional mutation. (PNG) [file pgen.1008336.s009.png]

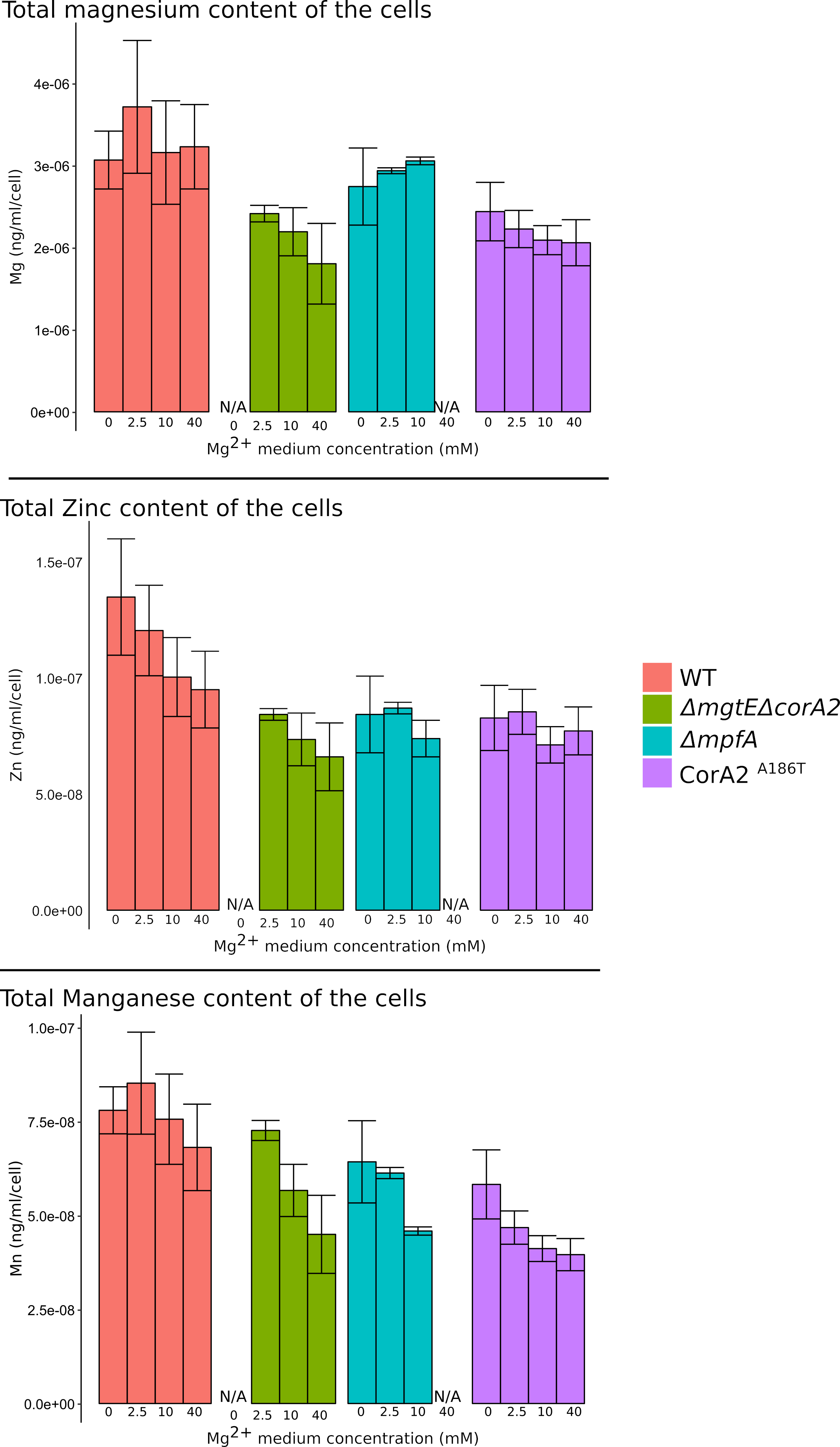

Supplement: S6 Fig — Total amounts of magnesium, manganese and zinc were quantified by ICP-OES (Inductively Coupled Plasma Optical Emission Spectrometry experiment) adapted from a previously described protocol (Arabet et al, 2014). Briefly, 109 bacterial cells of an exponential phase culture were harvested and lysed 10 min at 37°C with 1 mg/mL lysostaphin in 500μL PBS. The samples were wet washed with 32.5% nitric acid (Suprapur, Merck) for 12 h at 100°C (Neumann et al. 2009) and were then filled to a tenfold volume with water prior to inductively coupled plasma optical emission spectrometry (ICP-OES) analysis. Two replicates for each sample were carried out, and the average concentration values were calculated. Analysis was performed using a ThermoFisher ICAP 6000 ICP-OES. A multielement standard solution (Merck) was used as a reference. Bacterial cultures were grown in Mueller Hinton medium supplemented with the indicated amount of MgCl2. The conditions are identical to that of Fig 3. N/A indicates the concentration could not be determined since the strain does not grow in said condition. Calculation of mean and standard deviation were performed using the aggregate package in R. Arabet D, Tempel S, Fons M, Denis Y, Jourlin-Castelli C, Armitano J, et al. Effects of a sulfonylurea herbicide on the soil bacterial community. Environ Sci Pollut Res Int. 2014;21: 5619–5627. doi:10.1007/s11356-014-2512-9 (PNG) [file pgen.1008336.s010.png]

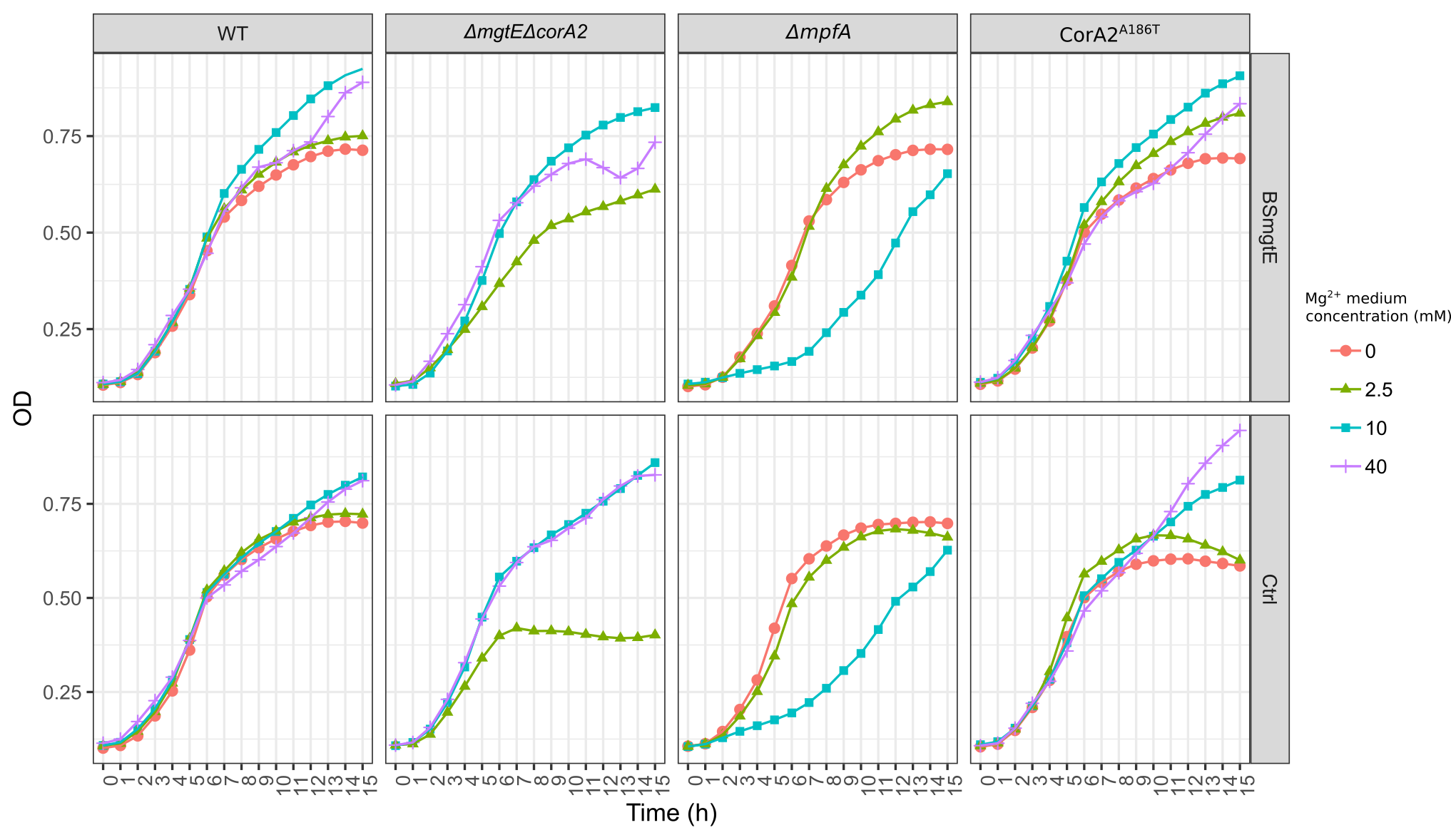

Supplement: S7 Fig — Bacteria where inoculated from overnight cultures at 1/100th and grown in a 96 well plate at 37°C under continuous agitation in Mueller Hinton medium (MH). OD600 was measured every hour. Growth of bacteria (OD) carrying the plasmid harboring the BSmgtE-GFP fusion (BSMgtE) or the constitutive promoter-GFP fusion (Ctrl) is plotted as a function of time. The BSmgtE and Ctrl samples where grown on the same day, in the same plate. (PNG) [file pgen.1008336.s011.png]

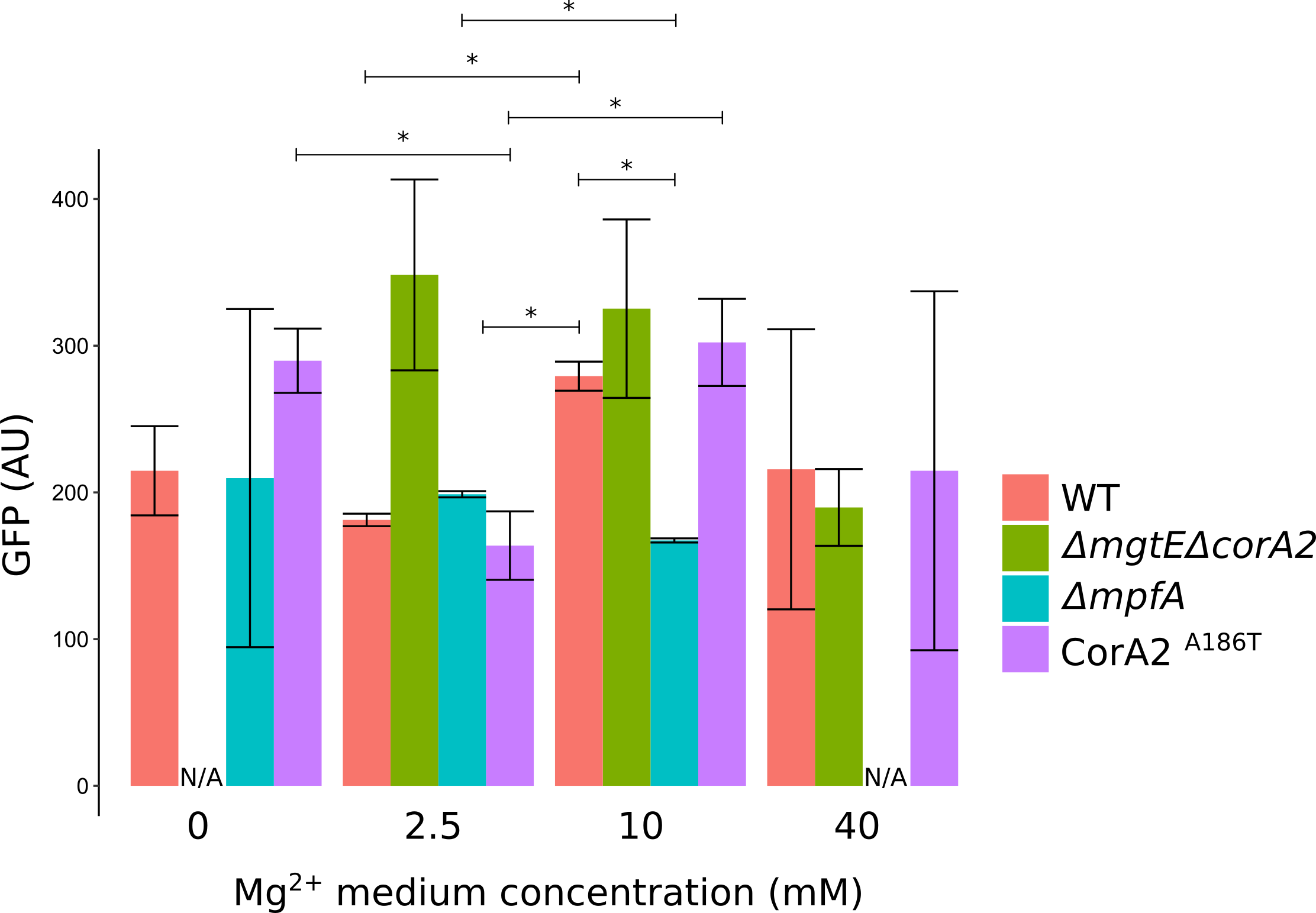

Supplement: S8 Fig — Fluorescence of cultures of different strains carrying a plasmid harboring a fusion between GFP and a constitutively expressed promoter (pHU) where measured mid-exponential phase. Bacteria where grown in MH medium supplemented with indicated amount of MgCl2. The value was calculated as the average of three independent measurements (N = 3), subtracted of the background noise, i.e. the inherent fluorescence of the medium. The results presented here are representative of at least three different experiments. The BSmgtE and Ctrl samples where grown on the same day, in the same plate. The significantly different results (* p-value<0.05) are shown. Although we observe some variations between strains and between external Mg concentrations, these only barely pass the threshold for statistical significance (p<0.05), and are far removed from the clear differences we observe in Fig 3. Unpaired t-test (R program) was used to calculate p-values. (PNG) [file pgen.1008336.s012.png]

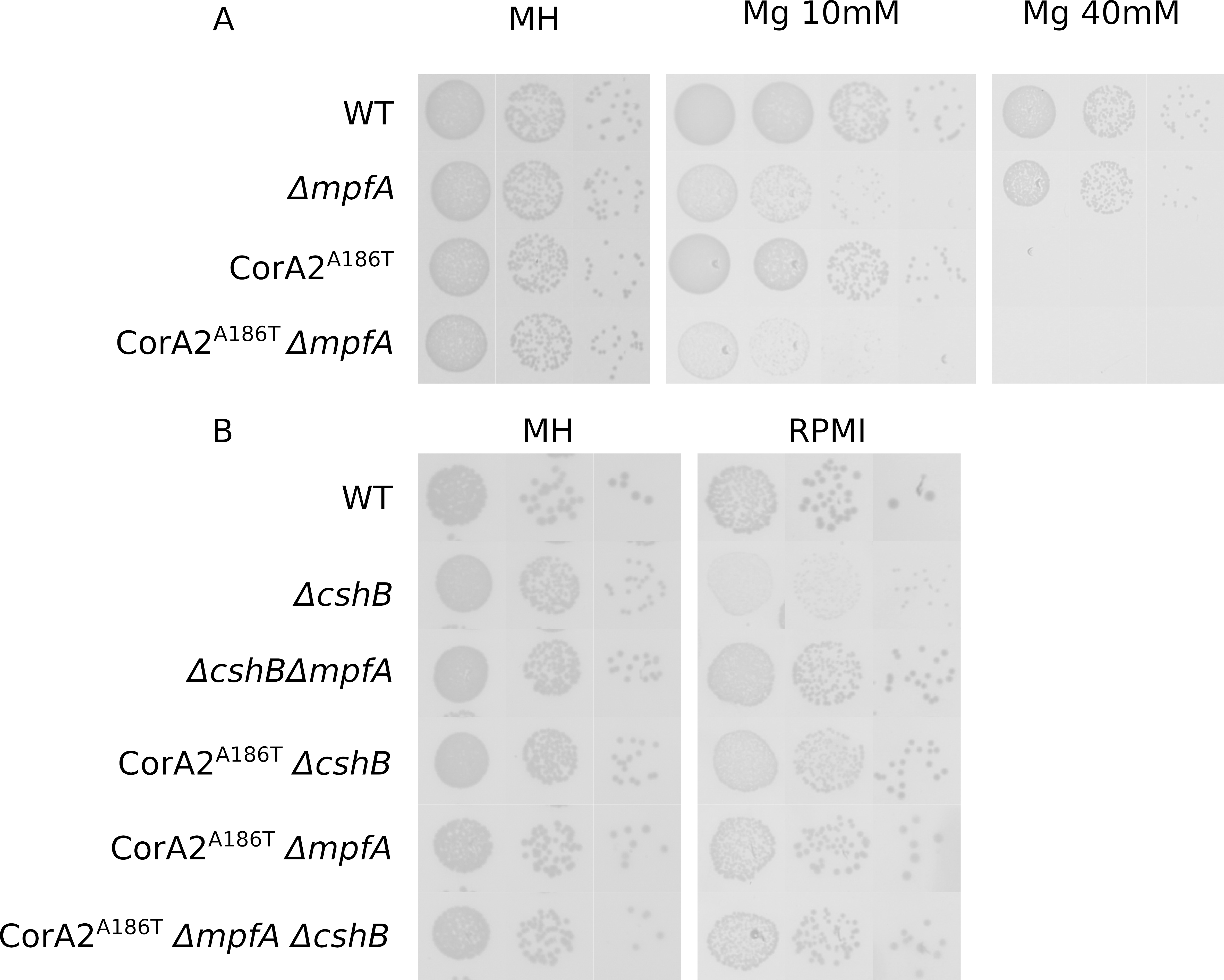

Supplement: S9 Fig — A: Dilutions of overnight cultures of each strain were spotted on Mueller Hinton medium (MH) supplemented with uracil and eventually the indicated amount of MgCl2. Plates were incubated for 24h at 37°C. B: Dilutions of overnight cultures of each strain were spotted on Mueller Hinton medium (MH) or RPMI medium supplemented with uracil. Plates were incubated for 24h at 37°C. (PNG) [file pgen.1008336.s013.png]

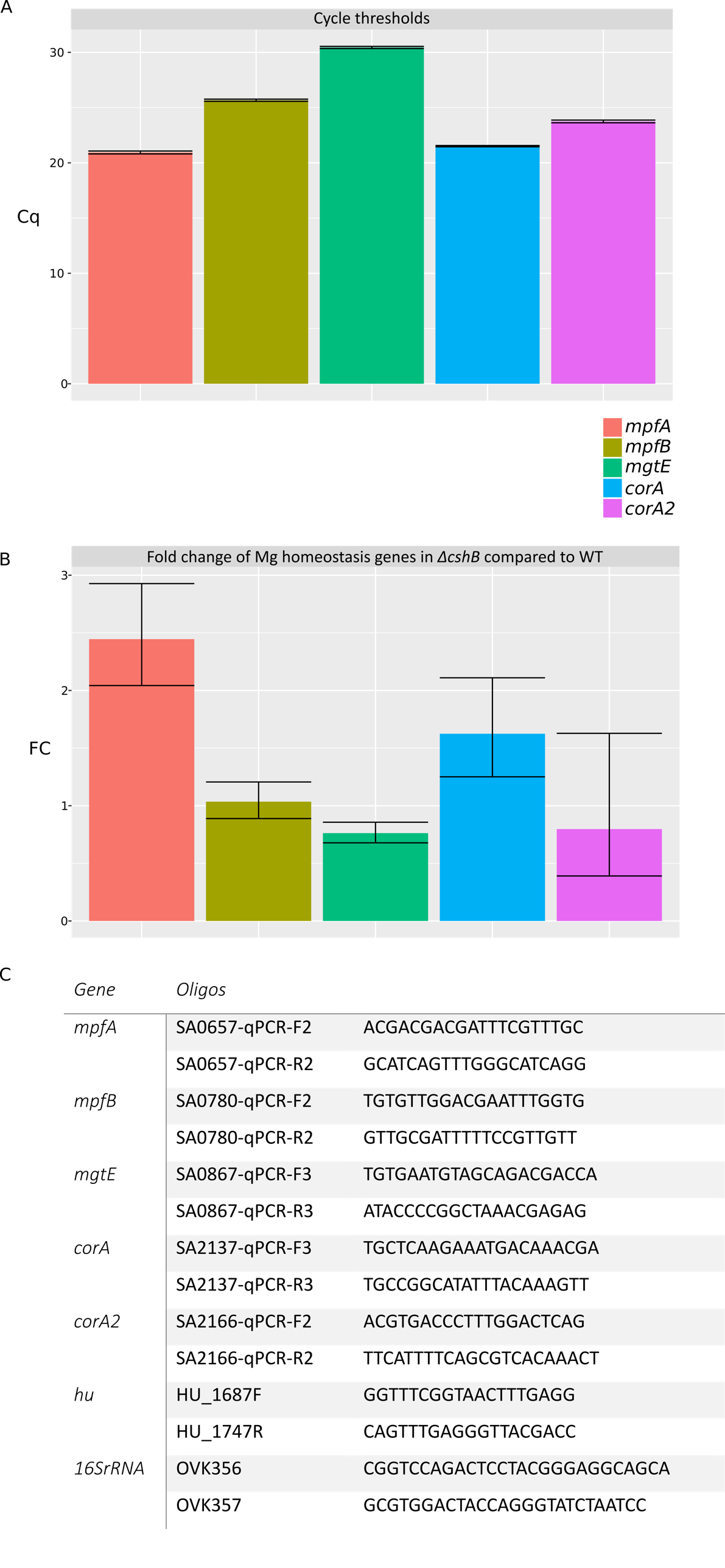

Supplement: S10 Fig — The RNA levels of the five genes implicated in magnesium homeostasis were measured by qRT-PCR. A: Graphical display of the cycle thresholds at which the different mRNAs were detected in a WT strain grown in absence of additional magnesium in MH medium at 37°C. All five RNAs can be detected albeit at varying levels. B: Comparison of expression of different targets between WT and ΔcshB strains, fold change compared to WT are plotted on the Y axis. C: qRT-PCR primers used in these experiments. (PNG) [file pgen.1008336.s014.png]
